# Iridium(I)-Catalyzed Isoindolinone-Directed Branched-Selective Aromatic C–H Alkylation with Simple Alkenes

**DOI:** 10.3390/molecules27061923

**Published:** 2022-03-16

**Authors:** Maoqian Xiong, Yuhang Shu, Jie Tang, Fan Yang, Dong Xing

**Affiliations:** Shanghai Engineering Research Center of Molecular Therapeutics and New Drug Development, School of Chemistry and Molecular Engineering, East China Normal University, Shanghai 200062, China; 18772300619@163.com (M.X.); shuyh2022@163.com (Y.S.); jtang@chem.ecnu.edu.cn (J.T.)

**Keywords:** iridium(I)-catalyzed, branched-selective, alkylation, *N*-arylisoindolinones, asymmetric

## Abstract

We report an iridium(I)-catalyzed branched-selective C–H alkylation of *N*-arylisoindolinones with simple alkenes as the alkylating agents. The amide carbonyl group of the isoindolinone motif acts as the directing group to assist the ortho C–H activation of the *N*-aryl ring. With this atom-economic and highly branched-selective protocol, an array of biologically relevant *N*-arylisoindolinones were obtained in good yields. Asymmetric control was achieved with up to 87:13 er when a BiPhePhos-like chiral ligand was employed.

## 1. Introduction

**Figure 1:**
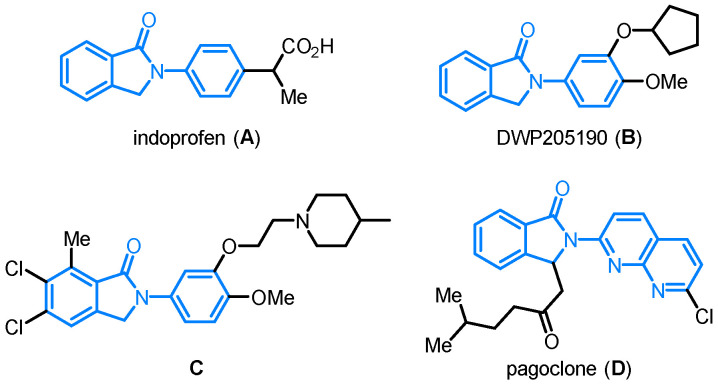
Representative examples of biologically active *N*-arylisoindolinones.

The skeleton of isoindolinone is widely present in a number of natural products, biologically active molecules and pharmaceuticals [1,2,3,4,5]. Among them, *N*-arylisoindolinones belong to an important class of compounds showing very broad biological activities [6,7]. For example, as shown in Figure 1, indoprofen (**A**) is known as a nonsteroidal anti-inflammatory drug (NSAID) and cyclo-oxygenase (COX) inhibitor [8], and DWP205190 (**B**) displays inhibitory activity toward tumor necrosis factor TNF-α production [9,10]. Compound **C** behaves as a potent and selective 5-HT_2C_ antagonist [11]. Pagoclone (**D**) is a partial benzodiazepine-GABA receptor agonist that is used for the treatment of panic and other anxiety disorders [12]. As a result, the development of new methodologies toward the rapid access of *N*-arylisoindolinones bearing different substituting patterns is highly appealing.

**Scheme 1:**
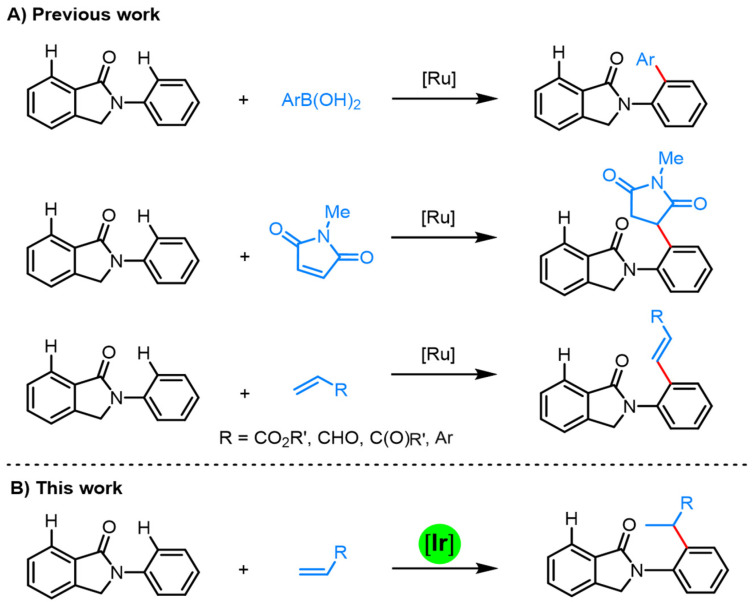
Isoindolinone-directed site-selective C–H activation. (**A**) Previous work; (**B**) This work.

Over the past decades, transition metal-catalyzed directing group (DG)-assisted aromatic C–H activation has emerged as a very powerful synthetic protocol [13,14,15,16,17,18,19,20], thus offering numerous direct C–H functionalization strategies for biologically related molecules. In this context, the amide-carbonyl group in the *N*-arylisoindolinone skeleton may act as a good directing group to assist the transition metal catalyst in the ortho position C–H activation. However, there are two distinct ortho C–H sites, either from the lactam-fused benzene ring or the *N*-aryl ring. As a result, the site-selective control is the key for realizing such type of C–H functionalizations. Recently, Gramage-Doria and coworkers successfully realized the site-selective C–H functionalization of *N*-arylisoindolinones by employing ruthenium catalysis. With aryl boronic acids, maleimides or activated alkenes such as α,β-unsaturated alkenes or styrenes as the functionalizing agents (Scheme 1A), the ortho C–H functionalization of the *N*-aryl ring was achieved with high efficiency [21,22,23]. Inspired by these advances, we decided to develop new types of C–H functionalization strategies by focusing on the biologically relevant *N*-arylisoindolinone motif.

In recent years, iridium catalysts have been successfully applied to an array of DG-assisted aromatic C–H alkylation reactions [24,25,26,27,28,29,30,31,32,33,34,35,36,37,38]. By employing alkenes or vinyl ethers as the alkylating agents, significant advances have been made by Shibata, Hartwig, Nishimura, Bower and others [26,27,28,29,30,31,32,33,34,35,36,37,38,39,40,41,42,43,44,45,46,47,48]. This iridium-catalyzed atom-economic C–H alkylation protocol generally favors the formation of branched-selective products [49,50]. By utilizing different types of chiral ligands, a number of asymmetric versions of such transformations have been realized [28,29,31,32,33,34,37,38]. In view of these progress, we envisioned that the amide carbonyl group of the isoindolinone motif may act as an efficient directing group, to enable the development of an iridium-catalyzed branched-selective C–H alkylation reaction with simple alkenes (Scheme 1B), and asymmetric control may be achieved with suitable chiral phosphine ligands. Herein, we report the details of this study. 

## 2. Results and Discussion

We chose *N*-phenylisoindolinone (**1a**) as the model substrate and 1-octene (**2a**) as the alkylating agent for our initial studies towards this iridium-catalyzed C–H functionalization reaction. To allow the rapid access of structurally diversified products without considering the stereochemistry for the initial biological evaluation purpose, racemic ligands were employed. Upon thorough condition optimizations in terms of the iridium sources, ligands, solvents and temperature, the desired branched-selective alkylation product **3a** was ultimately obtained in an 87% yield (isolated yield: 81%) with a >20:1 branched selectivity (Table 1, entry 1). A BiPhenPhos-like bidentate ligand (**L1**), of which the chiral form has been successfully utilized to the branched-selective and enantioselective *N*-acetyl directed C–H alkylation of anilides with alkenes as developed by Bower and coworkers [37], which was crucial for achieving the good reactivity as well as the excellent branched-selectivity control. The use of the cationic [Ir(COD)_2_]BArF pre-catalyst was also crucial for this transformation. A set of control experiments was conducted to understand the role of each reactant (Table 1). Among different bidentate phosphine ligands tested, *rac*-BINAP showed very low efficiency while *rac*-BIPHEP produced the desired product **3a** in a 12% yield with 10:1 branched selectivity (Table 1, entries 2 and 3). With dppf or dppb as the ligand, the desired product **3a** was afforded in high branched selectivities, albeit with 20–25% yields (entries 4 and 5). On the other hand, small bite-angle ligands such as dppe showed a low branched selectivity (entry 6). These results are in accordance with literature precedents in which the branched-selective product formation is favored by larger bite-angle ligands [35,36]. It is worth mentioning that for all these entries, no other side products (i.e., the *N*-aryl dialkylation product, alkylation product on the *ortho*-position on the aryl ring of the isoindolinone) were observed. Next, the effect of solvent was investigated. While different solvents such as cyclopentyl methyl ether (CPME), toluene, 1,2-dichloroethane (DCE), chlorobenzene or *m*-xylene could all produce the desired product in excellent branched selectivity, a decreased reaction efficiency was observed, and the use of 1,4-dioxane as the solvent was superior (Table 1, entries 7–11).

With the optimization conditions in hand, we applied this branched-selective C–H alkylation reaction toward the synthesis of different substituted *N*-arylisoindolinones (Table 2). Alkyl-substituted alkenes such as 1-octene or 1-hexene produced the corresponding products in high yields (**3a** and **3b**). Different styrene-type alkenes were then investigated and excellent branched selectivities were generally observed. Simple styrene or styrenes bearing electron-donating groups such as methyl or methoxyl at different positions were suitable substrates, yielding corresponding products with good efficiency (**3d–3g**). On the other hand, *para*- or *meta*-fluoro-substituted styrenes were less efficient, yielding the desired products in moderate yields (**3h** and **3i**) (56% or 58%, respectively). *N*-arylisoindolinones bearing different substituents on the *N*-aryl ring were then tested. Both electron-donating groups such methyl or methoxyl and electron-withdrawing groups such as chloro or fluoro at different positions could all be well-tolerated, giving the corresponding products in moderate to high yields (**3j–3p**). Again, excellent branched selectivities were observed for these substrates, thus guaranteeing the rapid collection of a set of *N*-arylisoindolinones with distinct electron properties. 

**Scheme 2:**
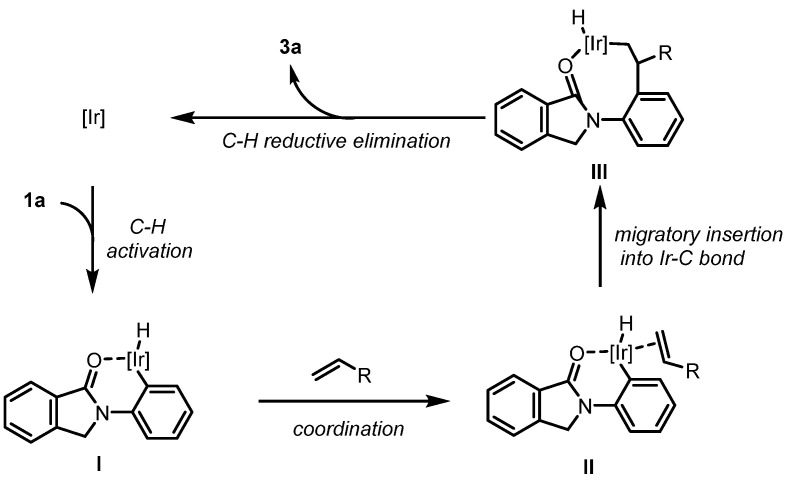
Plausible mechanism.

Based on our experimental results as well as literature precedents [37,49,50], a plausible mechanism by following a modified Chalk–Harrod type pathway is proposed for this iridium(I)-catalyzed branched-selective C–H alkylation reaction (Scheme 2). First, with the chelation assistance from the amide carbonyl group of the isoindolinone ring, the iridium catalyst activates the C–H bond by forming an iridium hydride species **I**. The alkene substrate then coordinates with intermediate **I** to form intermediate **II**. Migratory insertion of the alkene moiety into the Ir–C bond leads to intermediate **III**, by which the branched-selective product formation is favored. Intermediate **III** then undergoes C–H reductive elimination to deliver both the iridium(I) catalyst as well as the desired product **3a**. 

**Scheme 3:**
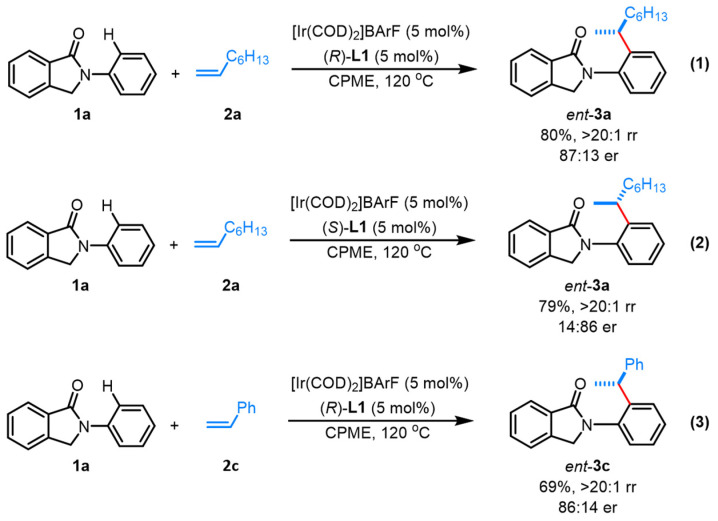
Asymmetric alkylation with chiral ligand.

With excellent branched-selective control being achieved, attempts for asymmetric control for this iridium-catalyzed alkylation were conducted with the screening of a handful of readily available chiral bidentate phosphine ligands. With 1-octene (**2a**) as the alkylating agents, a thorough condition optimization was conducted (see Supplementary Materials). Finally, with chiral BiPhePhos-like (*R*)-**L1** as the ligand and CPME as the solvent, the corresponding optically active product **3a** was obtained with excellent branched selectivity and high enantioselectivity (87:13 er) (Scheme 3, Equation (1)). Styrene (**2c**) was also applicable toward this asymmetric protocol, yielding corresponding product **3c** in 86:14 er (Scheme 3, Equation (3)). Furthermore, when (*S*)-**L1** was utilized as the ligand, the corresponding enantiomer of **3a** was obtained with 14:86 er (Scheme 3, Equation (2)), thus allowing the rapid access of both enantiomers of **3a** by switching the absolute configuration of the ligand. These results further illustrate the novel structural nature of the BiPhePhos-type ligand for the efficient asymmetric control on such types of alkylation reactions. This strategy also offers a powerful strategy for potential biological evaluation of structure-diversified chiral isoindolinone skeletons.

## 3. Experimental Section

Unless otherwise stated, ^1^H-NMRand ^13^C-NMRspectra were recorded on a Bruker (400 MHz) spectrometer. Chemical shifts were reported in parts per million (ppm), and the residual solvent peak was used as an internal reference: proton (chloroform δ 7.26), carbon (chloroform δ 77.0) or tetramethylsilane (TMS δ 0.00) was used as a reference. Data are reported as follows: chemical shift, multiplicity (s = singlet, d = doublet, t = triplet, dd = doublet of doublets, td = triplet of doublets, dt = doublet of triplets, ddd = doublet of doublet of doublets, m = multiplet, bs = broad singlet, etc.), coupling constants (Hz) and integration. High-resolution mass spectra (HRMS) were obtained on IonSpec FT-ICR or Waters Micromass Q-TOF micro Synapt high-definition mass spectrometer. Optical rotation was determined on RUDOLPH AUTOPOL-VI apparatus. Melting points were measured on INESA WRR-Y melting point apparatus. Flash chromatography was carried out with 300–400 mesh silica gel. All the key reactions were carried out under nitrogen atmosphere with a stir bar in a sealed vial. 1,4-dioxane (99.5%, extra dry, stabilized) used for the key reactions was purchased from Acros and degassed with nitrogen before use. [Ir(COD)_2_] BArF was prepared according to literature methods. All the ligands were purchased from Strem Chemicals and were used as received. All starting materials 1a–p were prepared according to literature methods [22,23,24].

### General Procedure for the Synthesis of Product (***3a***–***3p***)

In a N_2_-filled glovebox, a 4 mL baked vial charged with a stir bar and 6.4 mg of [Ir(COD)_2_]BArF (0.005 mmol), 4.0 mg of *rac*-**L1** (0.005 mmol) was added. Then, 0.2 mL of 1, 4-dioxane was added into the vial and the resulting solution was stirred for 5 min. Then, **1a** (21 mg, 0.1 mmol) and **2a** (56 mg, 0.5 mmol) were added. The vial was tightly capped with a screw cap and then removed from the glovebox and placed in a pre-heated aluminum block at 120 °C for 48 h. The reaction mixture was directly purified by column chromatography on silica gel with EtOAc/PE mixture as an eluent.

2-(2-(octan-2-yl)phenyl)isoindolin-1-one (**3a**)

White foam, 26.0 mg, 81% yield, ^1^H-NMR(400 MHz, CDCl_3_) δ 7.96 (d, *J* = 7.5 Hz, 1H), 7.64–7.58 (m, 1H), 7.56–7.50 (m, 2H), 7.41–7.38 (m, 2H), 7.30–7.25 (m, 1H), 7.21 (d, *J* = 7.6 Hz, 1H), 4.81–4.59 (m, 2H), 2.82–2.71 (m, 1H), 1.64–1.46 (m, 2H), 1.31–1.06 (m, 11H), 0.80 (t, *J* = 6.4 Hz, 3H). ^13^C-NMR(101 MHz, CDCl_3_) δ 167.28, 145.51, 140.45, 135.23, 131.41, 130.60, 127.75, 127.24, 127.02, 125.95, 125.62, 123.23, 121.73, 53.18, 37.05, 32.72, 30.65, 28.37, 26.80, 21.59, 21.28, 13.00. HRMS (ESI) calcd. for C_22_H_28_NO [M + H]^+^: 322.2081, Found: 322.2076. Chiralcel IC column, *n*-hexane/*i*-PrOH = 100:10, flow rate = 1.0 mL/min, λ = 254 nm, *t*_R_ (major isomer) = 25.168 min, *t*_R_ (minor isomer) = 21.322 min. er: 87:13.

2-(2-(hexan-2-yl)phenyl)isoindolin-1-one (**3b**) 

White foam, 24.9 mg, 85% yield, ^1^H-NMR(400 MHz, CDCl_3_) δ 7.89 (d, *J* = 7.4 Hz, 1H), 7.58–7.51 (m, 1H), 7.49–7.42 (m, 2H), 7.34–7.30 (m, 2H), 7.23–7.20 (m, 1H), 7.14 (d, *J* = 7.6 Hz, 1H), 4.77–4.51 (m, 2H), 2.79–2.62 (m, 1H), 1.61–1.40 (m, 2H), 1.25–0.95 (m, 7H), 0.73 (t, *J* = 7.0 Hz, 3H). ^13^C-NMR(101 MHz, CDCl_3_) δ 167.28, 145.50, 140.46, 135.25, 131.44, 130.59, 127.78, 127.26, 127.04, 125.97, 125.65, 123.29, 121.73, 53.19, 36.73, 32.79, 29.05, 21.77, 21.43, 12.92. HRMS (ESI) calcd. for C_20_H_24_NO [M + H]^+^: 294.1706, Found: 294.1700.

2-(2-(1-phenylethyl)phenyl)isoindolin-1-one (**3c**) 

White solid, 22.5 mg, 72% yield, m.p. = 130–132 °C. ^1^H-NMR(400 MHz, CDCl_3_) δ 7.96 (d, *J* = 7.1 Hz, 1H), 7.59–7.48 (m, 3H), 7.45–7.38 (m, 1H), 7.36–7.27 (m, 2H), 7.17 (d, *J* = 7.7 Hz, 1H), 7.05 (s, 3H), 6.95 (s, 2H), 4.54–4.31 (m, 2H), 3.53 (s, 1H), 1.61 (d, *J* = 7.0 Hz, 3H). ^13^C-NMR(101 MHz, CDCl_3_) δ 167.99, 146.41, 144.76, 141.97, 136.86, 132.21, 131.55, 128.59, 128.24, 128.22, 128.13, 127.93, 127.38, 127.35, 125.87, 124.13, 122.53, 53.40, 39.77, 21.85. HRMS (ESI) calcd. for C_22_H_20_NO [M+H]^+^: 314.1514, Found: 314.1562. Chiralcel IC column, *n*-hexane/*i*-PrOH = 100:10, flow rate = 1.0 mL/min, λ = 254 nm, *t*_R_ (major isomer) = 19.801 min, *t*_R_ (minor isomer) = 20.807 min. er: 86:14.

2-(2-(1-(p-tolyl)ethyl)phenyl)isoindolin-1-one (**3d**)

White solid, 28.4 mg, 87% yield, m.p. = 140–142 °C. ^1^H-NMR(400 MHz, CDCl_3_) δ 7.96 (d, *J* = 7.2 Hz, 1H), 7.60–7.48 (m, 3H), 7.43–7.37 (m, 1H), 7.35–7.28 (m, 2H), 7.17 (d, *J* = 7.6 Hz, 1H), 6.87 (s, 4H), 4.44 (d, *J* = 16.9 Hz, 1H), 4.34 (q, *J* = 6.9 Hz, 1H), 3.67 (s, 1H), 2.27 (s, 3H), 1.59 (d, *J* = 7.0 Hz, 3H). ^13^C-NMR(101 MHz, CDCl_3_) δ 167.00, 143.95, 142.27, 140.94, 135.70, 134.31, 131.21, 130.50, 127.83, 127.57, 127.18, 127.08, 126.93, 126.23, 126.18, 123.11, 121.45, 52.48, 38.22, 20.89, 19.88. HRMS (ESI) calcd. for C_23_H_22_NO [M + H]^+^: 328.1701, Found: 328.1708. 

2-(2-(1-(4-methoxyphenyl)ethyl)phenyl)isoindolin-1-one (**3e**) 

White solid, 29.8 mg, 87% yield, m.p. = 96–98 °C. ^1^H-NMR(400 MHz, CDCl_3_) δ 7.97 (d, *J* = 7.3 Hz, 1H), 7.60–7.46 (m, 3H), 7.43–7.37 (m, 1H), 7.36–7.28 (m, 2H), 7.17 (d, *J* = 7.7 Hz, 1H), 6.88 (s, 2H), 6.62 (d, *J* = 7.9 Hz, 2H), 4.45 (d, *J* = 16.9 Hz, 1H), 4.34 (q, *J* = 7.0 Hz, 1H), 3.70 (s, 4H), 1.58 (d, *J* = 7.1 Hz, 3H). ^13^C-NMR(101 MHz, CDCl_3_) δ 166.93, 156.63, 143.99, 140.89, 137.42, 135.67, 131.22, 130.50, 127.55, 127.21, 127.19, 127.09, 126.81, 126.22, 123.08, 121.51, 112.53, 54.17, 52.47, 37.79, 20.96. HRMS (ESI) calcd. for C_23_H_22_NO_2_ [M + H]^+^: 344.1651, Found: 344.1679. 

2-(2-(1-(2-methoxyphenyl)ethyl)phenyl)isoindolin-1-one (**3f**) 

White solid, 25.7 mg, 75% yield, m.p. = 157–159 °C. ^1^H-NMR(400 MHz, CDCl_3_) δ 7.96 (d, *J* = 7.1 Hz, 1H), 7.61–7.47 (m, 3H), 7.44–7.37 (m, 1H), 7.33–7.27 (m, 2H), 7.14 (d, *J* = 7.6 Hz, 1H), 7.08–6.98 (m, 1H), 6.88 (d, *J* = 7.4 Hz, 1H), 6.82–6.74 (m, 1H), 6.47 (d, *J* = 8.1 Hz, 1H), 4.84 (q, *J* = 6.9 Hz, 1H), 4.35 (d, *J* = 16.9 Hz, 1H), 3.42 (d, *J* = 16.8 Hz, 1H), 3.10 (s, 3H), 1.54 (d, *J* = 7.0 Hz, 3H). ^13^C-NMR(101 MHz, CDCl_3_) δ 167.94, 156.09, 145.51, 142.09, 136.87, 135.06, 132.60, 131.21, 128.42, 128.30, 128.12, 127.86, 127.60, 127.08, 126.82, 124.03, 122.42, 120.29, 109.77, 54.38, 52.95, 31.96, 20.53. HRMS (ESI) calcd. for C_23_H_22_NO_2_ [M + H]^+^: 344.1651, Found: 344.1679. 

2-(2-(1-(m-tolyl)ethyl)phenyl)isoindolin-1-one (**3g**) 

White solid, 23.5 mg, 72% yield, m.p. = 119–121 °C. ^1^H-NMR(400 MHz, CDCl_3_) δ 7.97 (d, *J* = 6.7 Hz, 1H), 7.60–7.48 (m, 3H), 7.46–7.38 (m, 1H), 7.35–7.24 (m, 2H), 7.16 (d, *J* = 7.3 Hz, 1H), 6.98–6.92 (m, 1H), 6.83 (d, *J* = 6.6 Hz, 1H), 6.70 (s, 2H), 4.45–4.08 (m, 2H), 3.34 (s, 1H), 1.94 (s, 3H), 1.59 (d, *J* = 6.9 Hz, 3H). ^13^C-NMR(101 MHz, CDCl_3_) δ 167.86, 146.42, 144.73, 142.07, 137.89, 136.92, 132.32, 131.48, 128.52, 128.39, 128.25, 128.07, 128.05, 127.72, 127.31, 126.49, 124.17, 124.11, 122.43, 53.31, 39.92, 21.72, 21.00. HRMS (ESI) calcd. for C_23_H_22_NO [M + H]^+^: 328.1701, Found: 328.1708. 

2-(2-(1-(4-fluorophenyl)ethyl)phenyl)isoindolin-1-one (**3h**) 

White solid, 19.2 mg, 58% yield, m.p. = 134–136 °C. ^1^H-NMR(400 MHz, CDCl_3_) δ 7.96 (d, *J* = 7.2 Hz, 1H), 7.66–7.28 (m, 6H), 7.18 (d, *J* = 7.5 Hz, 1H), 6.93 (s, 2H), 6.84–6.70 (m, 2H), 4.56–4.28 (m, 2H), 3.65 (s, 1H), 1.58 (d, *J* = 7.0 Hz, 3H). ^13^C-NMR(101 MHz, CDCl_3_) δ 167.94, 161.09 (d, *J* = 244.1 Hz), 144.57, 142.04, 141.78, 136.74, 132.15, 131.69, 128.77, 128.69, 128.66, 128.24, 127.88, 127.50, 124.16, 122.60, 114.93 (d, *J* = 21.1 Hz), 53.47, 38.94, 21.99. HRMS (ESI) calcd. for C_22_H_19_FNO [M + H]^+^: 332.1451, Found: 332.1478.

2-(2-(1-(3-fluorophenyl)ethyl)phenyl)isoindolin-1-one (**3i**) 

White solid, 18.5 mg, 56% yield, m.p. = 121–123 °C. ^1^H-NMR(400 MHz, CDCl_3_) δ 7.96 (d, *J* = 7.4 Hz, 1H), 7.59–7.38 (m, 4H), 7.37–7.31 (m, 2H), 7.19 (d, *J* = 7.7 Hz, 1H), 7.04–6.97 (m, 1H), 6.79–6.65 (m, 3H), 4.49 (d, *J* = 16.8 Hz, 1H), 4.41 (q, *J* = 7.1 Hz, 1H), 3.64 (s, 1H), 1.59 (d, *J* = 7.1 Hz, 3H). ^13^C-NMR(101 MHz, CDCl_3_) δ 167.96, 162.77 (d, *J* = 245.8 Hz), 149.14, 144.08, 141.76, 136.80, 132.10, 131.70, 129.61 (d, *J* = 8.2 Hz), 128.70, 128.26, 128.22, 127.94, 127.63, 124.18, 123.20, 122.56, 114.16 (d, *J* = 21.4 Hz), 112.77 (d, *J* = 21.2 Hz), 53.45, 39.46, 21.70. HRMS (ESI) calcd. for C_22_H_19_FNO [M + H]^+^: 332.1451, Found: 332.1478.

2-(4-methyl-2-(1-phenylethyl)phenyl)isoindolin-1-one (**3j**) 

White solid, 20.3 mg, 62% yield, m.p. = 146–148 °C. ^1^H-NMR(400 MHz, CDCl_3_) δ 7.96 (d, *J* = 6.9 Hz, 1H), 7.59–7.47 (m, 2H), 7.36–7.28 (m, 2H), 7.17–6.93 (m, 7H), 4.48–4.24 (m, 2H), 3.53 (s, 1H), 2.42 (s, 3H), 1.61 (d, *J* = 7.1 Hz, 3H). ^13^C-NMR(101 MHz, CDCl_3_) δ 168.09, 146.53, 144.30, 141.99, 138.39, 134.20, 132.32, 131.47, 128.62, 128.21, 128.07, 128.04, 127.99, 127.37, 125.83, 124.09, 122.52, 53.43, 39.68, 21.87, 21.52. HRMS (ESI) calcd. for C_23_H_22_NO [M + H]^+^: 328.1701, Found: 328.1708. 

2-(4-methoxy-2-(1-phenylethyl)phenyl)isoindolin-1-one (**3k**) 

White solid, 27.4 mg, 80% yield, m.p. = 140–142 °C. ^1^H-NMR(400 MHz, CDCl_3_) δ 7.94–7.83 (m, 1H), 7.50–7.39 (m, 2H), 7.21 (s, 1H), 7.05–6.74 (m, 8H), 4.34–4.21 (m, 2H), 3.77 (s, 3H), 3.29 (s, 1H), 1.51 (d, *J* = 7.1 Hz, 3H). ^13^C-NMR(101 MHz, CDCl_3_) δ 168.22, 159.53, 146.09, 141.97, 134.83, 132.26, 131.48, 129.63, 129.23, 128.25, 128.06, 127.32, 125.92, 124.07, 122.50, 114.30, 111.65, 55.50, 53.52, 39.94, 21.84. HRMS (ESI) calcd. for C_23_H_22_NO_2_ [M + H]^+^: 344.1651, Found: 344.1679.

2-(4-fluoro-2-(1-phenylethyl)phenyl)isoindolin-1-one (**3l**) 

White solid, 27.1 mg, 82% yield, m.p. = 129–131 °C. ^1^H-NMR(400 MHz, CDCl_3_) δ 7.87 (d, *J* = 7.1 Hz, 1H), 7.52–7.41 (m, 2H), 7.25–6.81 (m, 9H), 4.46–4.21 (m, 2H), 3.37 (s, 1H), 1.50 (d, *J* = 7.1 Hz, 3H). ^13^C-NMR(101 MHz, CDCl_3_) δ 167.03, 161.45 (d, *J* = 247.4 Hz), 146.34, 144.57, 140.83, 131.69, 130.91, 130.64, 128.80 (d, *J* = 9.0 Hz), 127.30, 127.15, 126.21, 125.10, 123.10, 121.50, 114.00 (d, *J* = 22.7 Hz), 113.09 (d, *J* = 22.6 Hz), 52.32, 38.87, 20.69. HRMS (ESI) calcd. for C_22_H_19_FNO [M + H]^+^: 332.1451, Found: 332.1478. 

2-(5-methyl-2-(1-phenylethyl)phenyl)isoindolin-1-one (**3m**) 

White solid, 19.6 mg, 60% yield, m.p. = 157–159 °C. ^1^H-NMR(400 MHz, CDCl_3_) δ 7.96 (d, *J* = 7.1 Hz, 1H), 7.60–7.46 (m, 2H), 7.39 (d, *J* = 4.5 Hz, 1H), 7.33–7.18 (m, 2H), 7.13–6.89 (m, 6H), 4.51–4.21 (m, 2H), 3.53 (s, 1H), 2.34 (s, 3H), 1.59 (d, *J* = 7.1 Hz, 3H). ^13^C-NMR(101 MHz, CDCl_3_) δ 167.02, 145.58, 140.91, 140.59, 136.14, 135.52, 131.21, 130.46, 128.34, 127.79, 127.15, 127.05, 126.74, 126.27, 124.75, 123.07, 121.46, 52.35, 38.41, 20.86, 19.77. HRMS (ESI) calcd. for C_23_H_22_NO [M + H]^+^: 328.1701, Found: 328.1708. 

2-(5-methoxy-2-(1-phenylethyl)phenyl)isoindolin-1-one (**3n**) 

White solid, 28.1 mg, 82% yield, m.p. = 170–172 °C. ^1^H-NMR(400 MHz, CDCl_3_) δ 7.95 (d, *J* = 6.3 Hz, 1H), 7.59–7.47 (m, 2H), 7.41 (d, *J* = 7.3 Hz, 1H), 7.28 (d, *J* = 6.1 Hz, 1H), 7.08–6.88 (m, 6H), 6.72 (s, 1H), 4.49–4.22 (m, 2H), 3.79 (s, 3H), 3.55 (s, 1H), 1.58 (d, *J* = 6.4 Hz, 3H). ^13^C-NMR(101 MHz, CDCl_3_) δ 167.93, 158.65, 146.79, 141.92, 137.57, 136.83, 132.16, 131.57, 128.64, 128.18, 128.13, 127.25, 125.79, 124.13, 122.54, 114.12, 113.79, 55.45, 53.32, 39.18, 22.02. HRMS (ESI) calcd. for C_23_H_22_NO_2_ [M + H]^+^: 344.1651, Found: 344.1679. 

2-(5-chloro-2-(1-phenylethyl)phenyl)isoindolin-1-one (**3o**) 

White solid, 16.7 mg, 48% yield, m.p. = 169–172 °C. ^1^H-NMR(400 MHz, CDCl_3_) δ 7.88 (d, *J* = 7.2 Hz, 1H), 7.55–7.41 (m, 2H), 7.41–7.30 (m, 2H), 7.22 (d, *J* = 7.1 Hz, 1H), 7.11 (s, 1H), 6.98 (s, 3H), 6.84 (s, 2H), 4.39–4.22 (m, 2H), 3.43 (s, 1H), 1.51 (d, *J* = 7.1 Hz, 3H). ^13^C-NMR(101 MHz, CDCl_3_) δ 166.83, 144.86, 142.50, 140.79, 136.96, 131.42, 130.76, 130.74, 128.03, 127.62, 127.43, 127.27, 127.22, 126.20, 125.03, 123.16, 121.54, 52.14, 38.45, 20.71. HRMS (ESI) calcd. for C_22_H_19_ClNO [M + H]^+^: 348.1155, Found: 348.1168. 

2-(5-fluoro-4-methyl-2-(1-phenylethyl)phenyl)isoindolin-1-one (**3p**) 

White solid, 20.7 mg, 61% yield, m.p. = 130–132 °C. ^1^H-NMR(400 MHz, CDCl_3_) δ 7.87 (d, *J* = 7.2 Hz, 1H), 7.52–7.39 (m, 2H), 7.24–7.19 (m, 2H), 6.98 (s, 3H), 6.85 (s, 2H), 6.77 (d, *J* = 9.6 Hz, 1H), 4.41–4.15 (m, 2H), 3.46 (s, 1H), 2.25 (s, 3H), 1.50 (d, *J* = 7.0 Hz, 3H). ^13^C-NMR(101 MHz, CDCl_3_) δ 166.96, 158.63 (d, *J* = 245.6 Hz), 145.26, 140.79, 139.20, 134.23 (d, *J* = 9.2 Hz), 130.89, 130.66, 129.45 (d, *J* = 5.7 Hz), 127.21, 127.15, 126.20, 124.89, 124.08 (d, *J* = 16.7 Hz), 123.13, 121.52, 113.85 (d, *J* = 22.7 Hz), 52.18, 38.20, 20.92, 13.64 (d, *J* = 2.9 Hz). HRMS (ESI) calcd. for C_23_H_21_FNO [M + H]^+^: 346.1607, Found: 346.1635. 

## 4. Conclusions

In summary, we have developed an iridium-catalyzed branched-selective C–H alkylation of *N*-arylisoindolinones with simple alkenes. With the assistance of the isoindolinone skeleton as a directing group, site-selective C–H activation was achieved at the ortho-position of the *N*-aryl ring. An array of biological *N*-arylisoindolinones were obtained in good yields and excellent branched selectivities. With a BiPhePhos-type chiral ligand, efficient asymmetric control was achieved with up to 87:13 er. The application of this methodology toward other types of biologically-relevant structures as well as medicinal studies of the resulted *N*-arylisoindolinones are currently ongoing.

## Figures and Tables

**Table 1:**
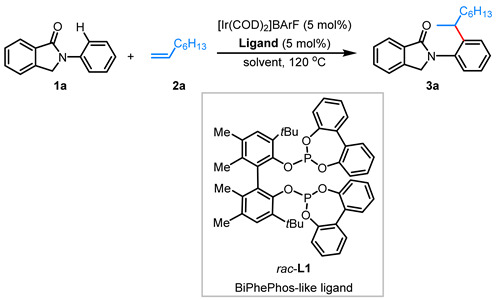
Condition optimizations ^a^.

Entry	Ligand (5 mol%)	Solvent	Yield (%) ^b^	rr (b/l) ^c^
1	*rac*-**L1**	1,4-dioxane	87 (81)	>20:1
2	*rac*-BINAP	1,4-dioxane	<5	--
3	*rac*-BIPHEP	1,4-dioxane	12	10:1
4	dppf	1,4-dioxane	25	11:1
5	dppb	1,4-dioxane	20	>20:1
6	dppe	1,4-dioxane	10	6:1
7	*rac*-**L1**	CPME	78	>20:1
8	*rac*-**L1**	toluene	51	>20:1
9	*rac*-**L1**	DCE	48	>20:1
10	*rac*-**L1**	PhCl	46	>20:1
11	*rac*-**L1**	*m*-xylene	63	>20:1

^a^ Reaction conditions: **1a** (0.1 mmol), **2a** (0.5 mmol,), [Ir(COD)_2_]BArF (5 mol%), ligand (5 mol%), solvent (0.2 mL), 120 °C, 48 h, ^b^ Yield was determined by GC analysis of the crude reaction mixture with dodecane as internal standard; isolated yield shown in parentheses. ^c^ The branched/linear (b/l) ratio was determined by GC analysis of the crude reaction mixture.

**Table 2:**
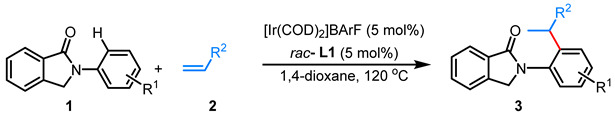
Substrate scope (**3a–p**) ^a^.

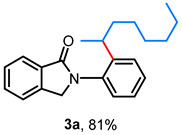	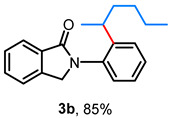	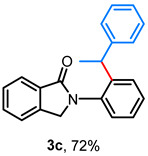	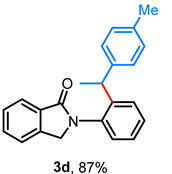
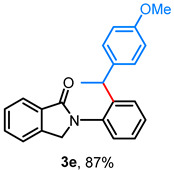	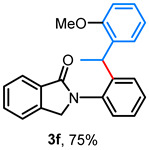	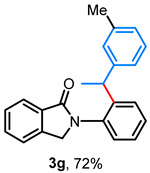	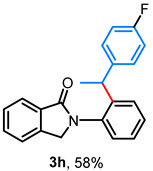
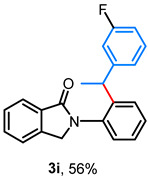	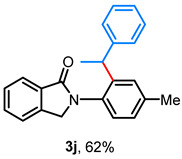	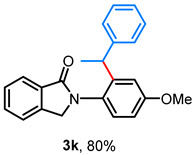	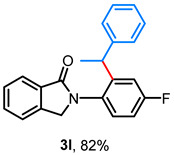
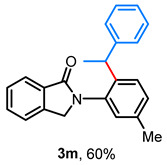	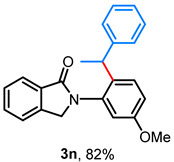	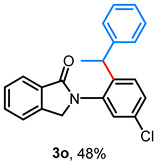	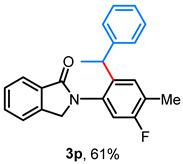

^a^ Reaction conditions: **1** (0.1 mmol), **2** (0.5 mmol,), [Ir(COD)_2_]BArF (5 mol%), *rac*-**L1** (5 mol%), 1,4-dioxane (0.2 mL), 120 °C, 48 h. Isolated yield for branched-selective product. The (b/l) ratio was >20:1 for all.

## Data Availability

The date presents in study are available in Supplementary Materials.

## Supplementary Materials

The following supporting information can be downloaded at: https://www.mdpi.com/article/10.3390/molecules27061923/s1, The chiral ligand screening, ^1^H-NMR and ^13^C-NMRspectra for all new compounds. HPLC spectra for **3a** and **3c**.

## Sample Availability

Samples of the compounds are not available from the authors.
