# Supplementary material for: Iridium(I)-Catalyzed Isoindolinone-Directed Branched-Selective Aromatic C–H Alkylation with Simple Alkenes
Source: Molecules. 2022 Mar 16;27(6):1923. doi: 10.3390/molecules27061923 (PMC8954050; doi:10.3390/molecules27061923)
Supplement: Supplementary file 1 [file molecules-27-01923-s001.zip › molecules-1620556-supplementary.pdf]

Supporting Information

Iridium (I)-Catalyzed Isoindolinone-Directed Branched-Selective Aromatic C–H Alkylation with Simple Alkenes

Maoqian Xiong, Yuhang Shu, Jie Tang, Fan Yang * and Dong Xing *

Shanghai Engineering Research Center of Molecular Therapeutics and New Drug Development, School of Chemistry and Molecular Engineering, East China Normal University, Shanghai 200062, China; 18772300619@163.com (M.X.); shuyh2022@163.com (Y.S.); jtang@chem.ecnu.edu.cn (J.T.)
* Correspondence: fyang@chem.ecnu.edu.cn (F.Y.); dxing@sat.ecnu.edu.cn (D.X.)

Table of Contents

1. Screening of Chiral Ligands and Condition Optimizations.. S2

2. NMR Spectra S3

3. HPLC Spectra S19

1. Screening of Chiral Ligands and Condition Optimizations[a]

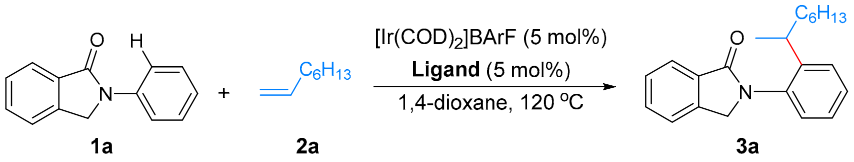

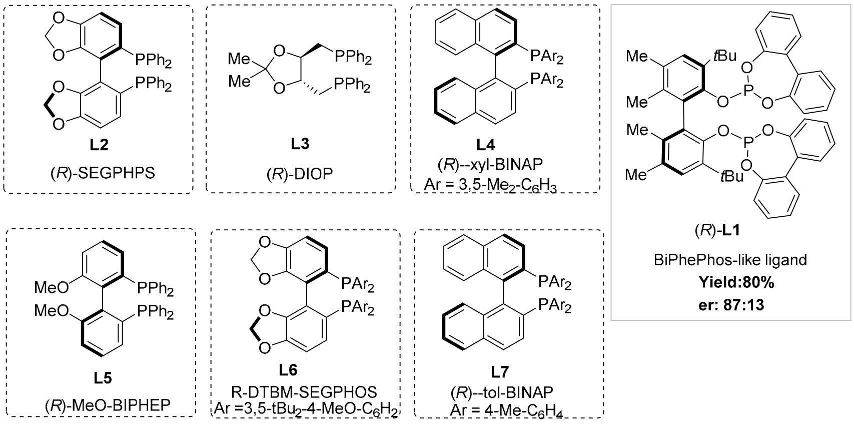

Entry	Ligand	solvent	Yield (%)[b]	er[c]	b/l[d]
1	**(*R*)-L1**	1,4-dioxane	81%	86:14	>20:1
2	**L2**	1,4-dioxane	21%	54:46	>20:1
3	**L3**	1,4-dioxane	56%	56:44	13:1
4	**L4**	1,4-dioxane	16%	65:35	18:1
5	**L5**	1,4-dioxane	42%	68:32	10:1
6	**L6**	1,4-dioxane	Trace		
7	**L7**	1,4-dioxane	11%	60:40	7:1
8	**(*R*)-L1**	CPME	80	87:13	> 20:1
9	**(*R*)- L1**	toluene	55	86:14	>20:1
10	**(*R*)- L1**	DCE	53	86:14	>20:1
11	**(*R*)- L1**	PhCl	61	86:14	>20:1
12	**(*R*)- L1**	*m*-xylene	60	86:14	>20:1
13	**(*R*)- L1**	DME	48	84:16	>20:1
14	**(*S*)-L1**	CPME	79	14:86	>20:1

[a]Reaction conditions: **1a** (0.1mmol), **2a** (0.5 mmol,), [Ir(COD)2]BArF (5 mol %), Ligand(5 mol%), solvent (0.2 mL), 120 °C, 48 h, [b]isolated yield for branched-selective product, [c]The er value of the product was determined by chiral HPLC analysis. [d]The linear/branched (l/b) ratio was determined by GC.

2. NMRSpectra

^{1}H NMR Spectrum of Compound **3a**

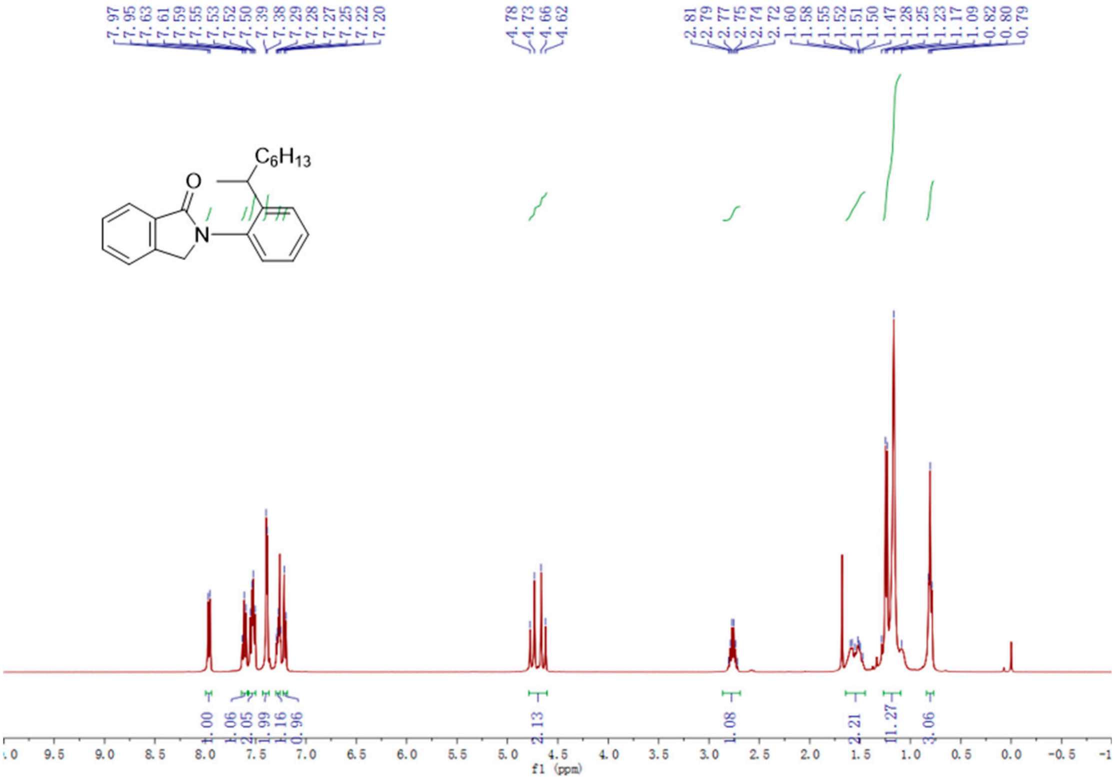

^{13}C NMR Spectrum of Compound **3a**

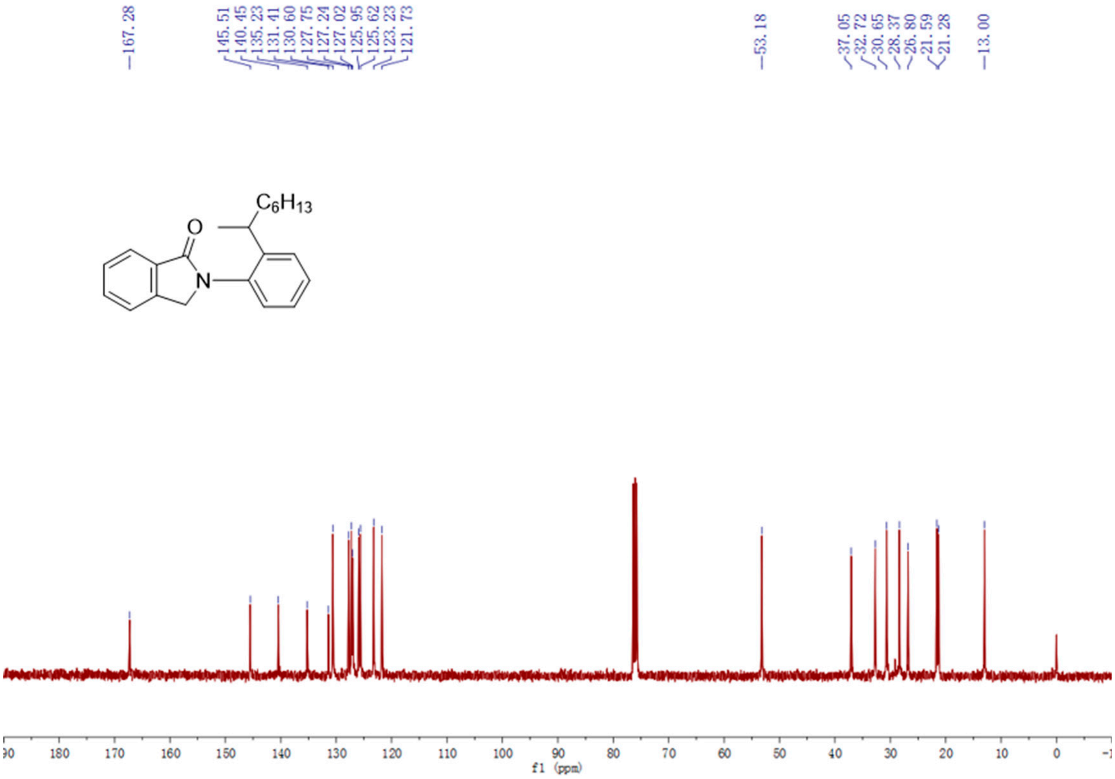

^{1}H NMR Spectrum of Compound **3b**

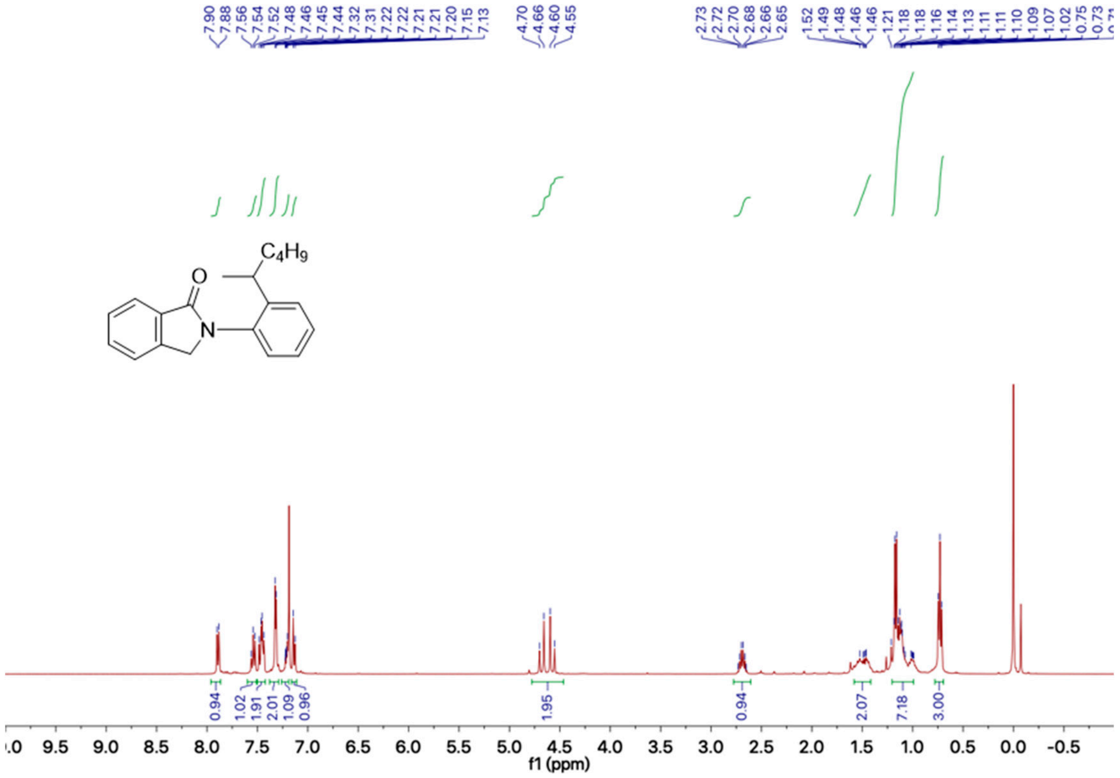

^{13}C NMR Spectrum of Compound **3b**

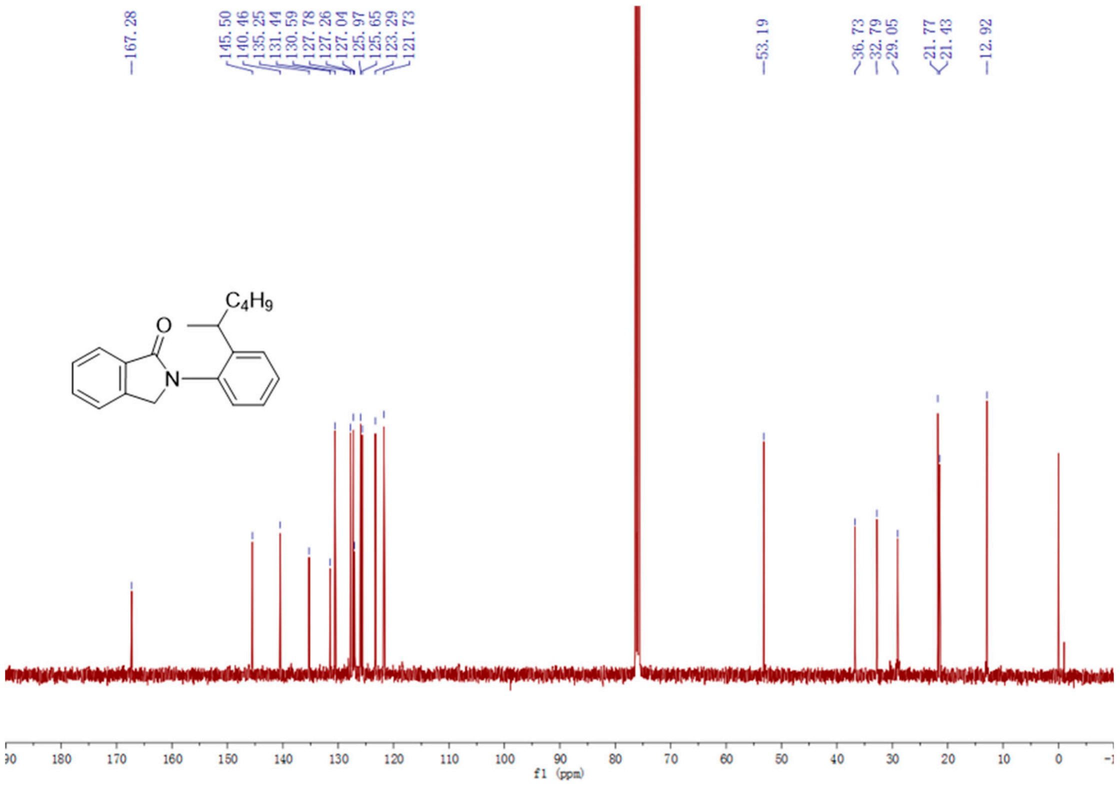

^{1}H NMR Spectrum of Compound **3c**

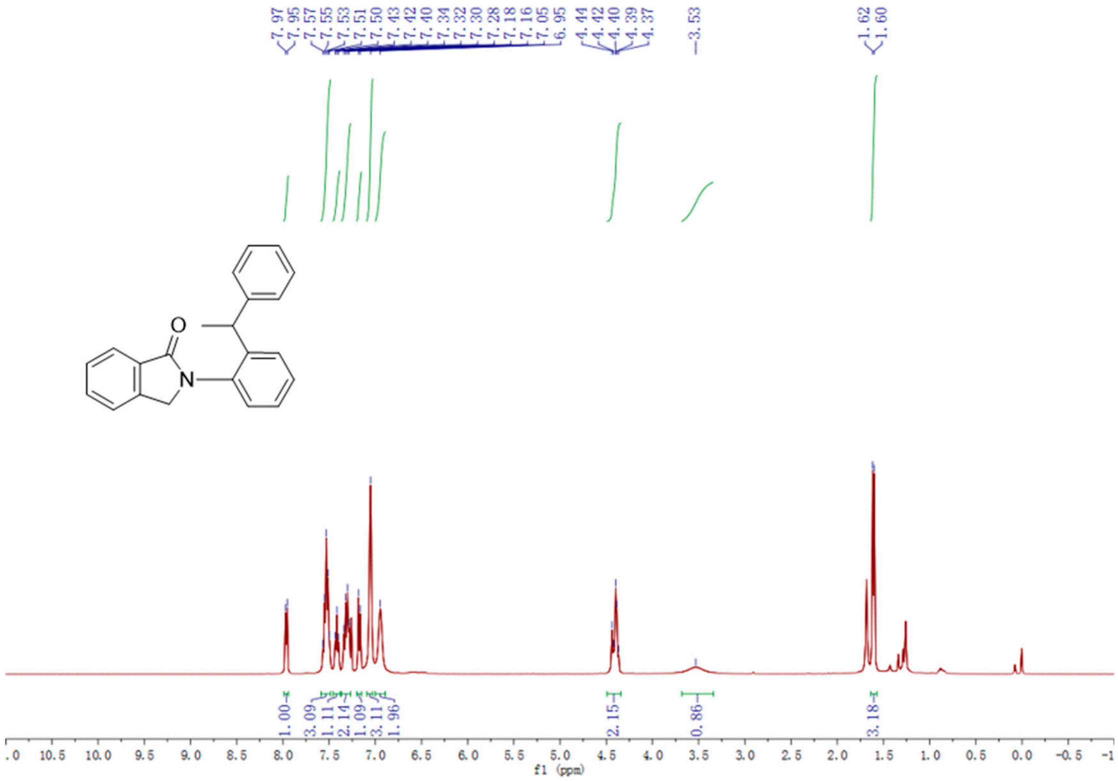

^{13}C NMR Spectrum of Compound **3c**

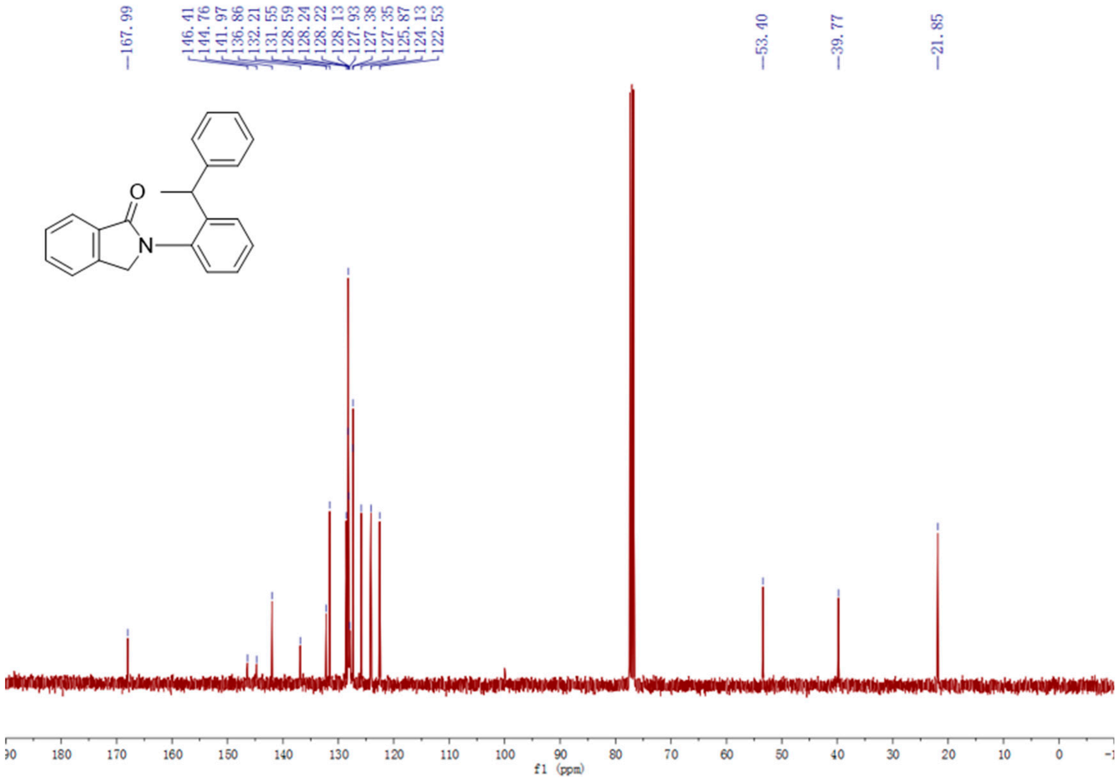

^{1}H NMR Spectrum of Compound **3d**

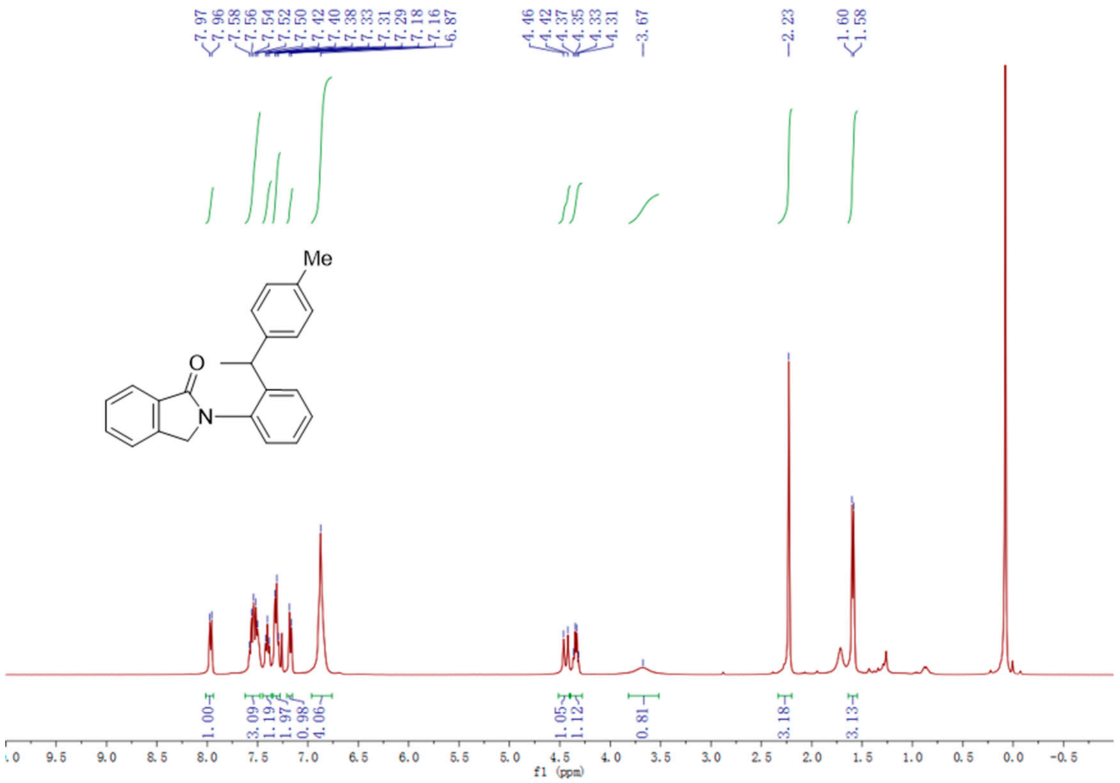

^{13}C NMR Spectrum of Compound **3d**

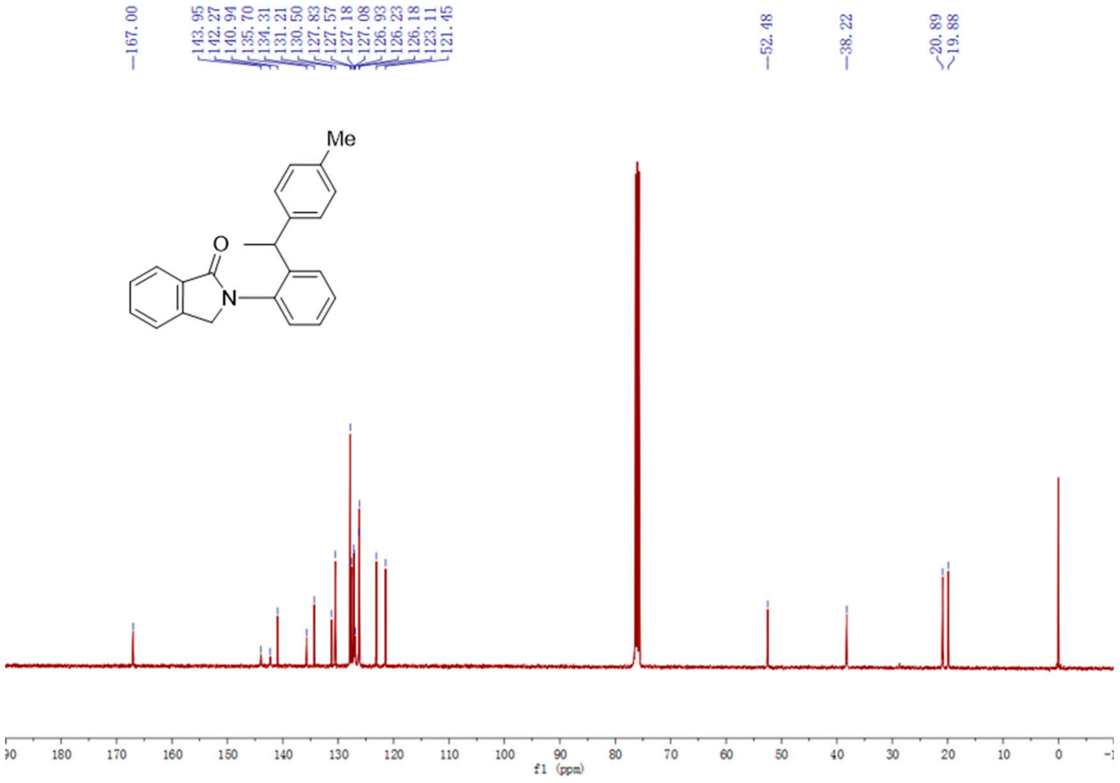

^{1}H NMR Spectrum of Compound **3e**

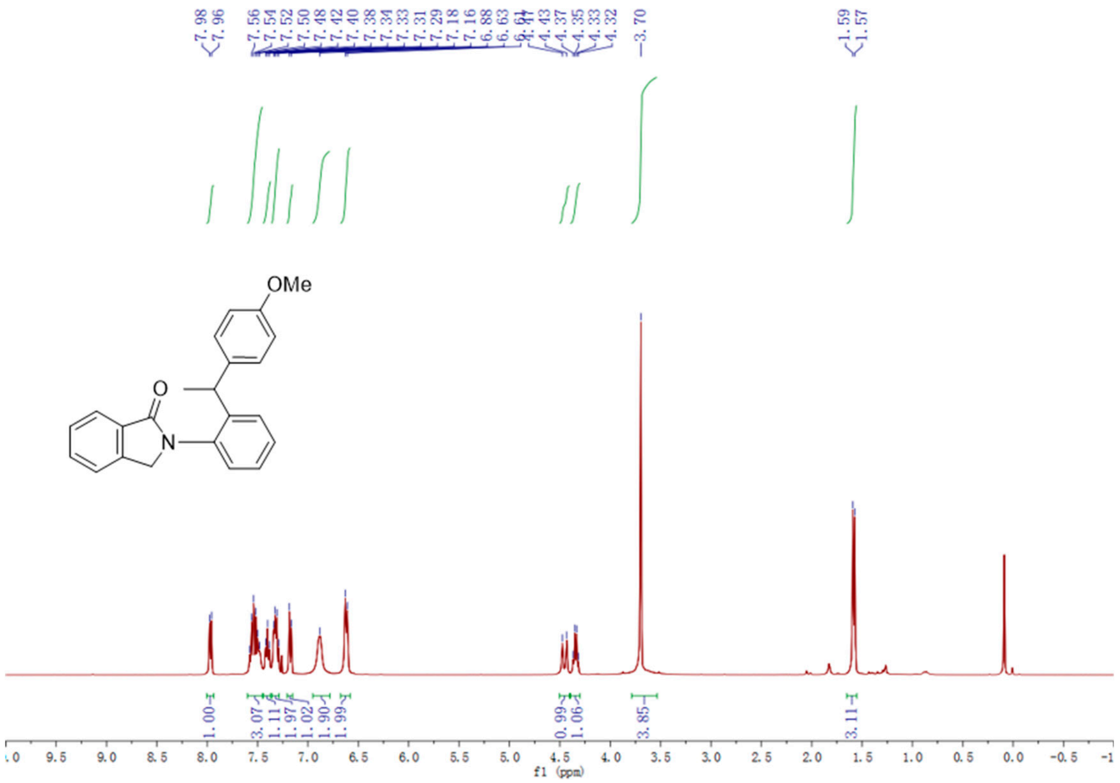

^{13}C NMR Spectrum of Compound **3e**

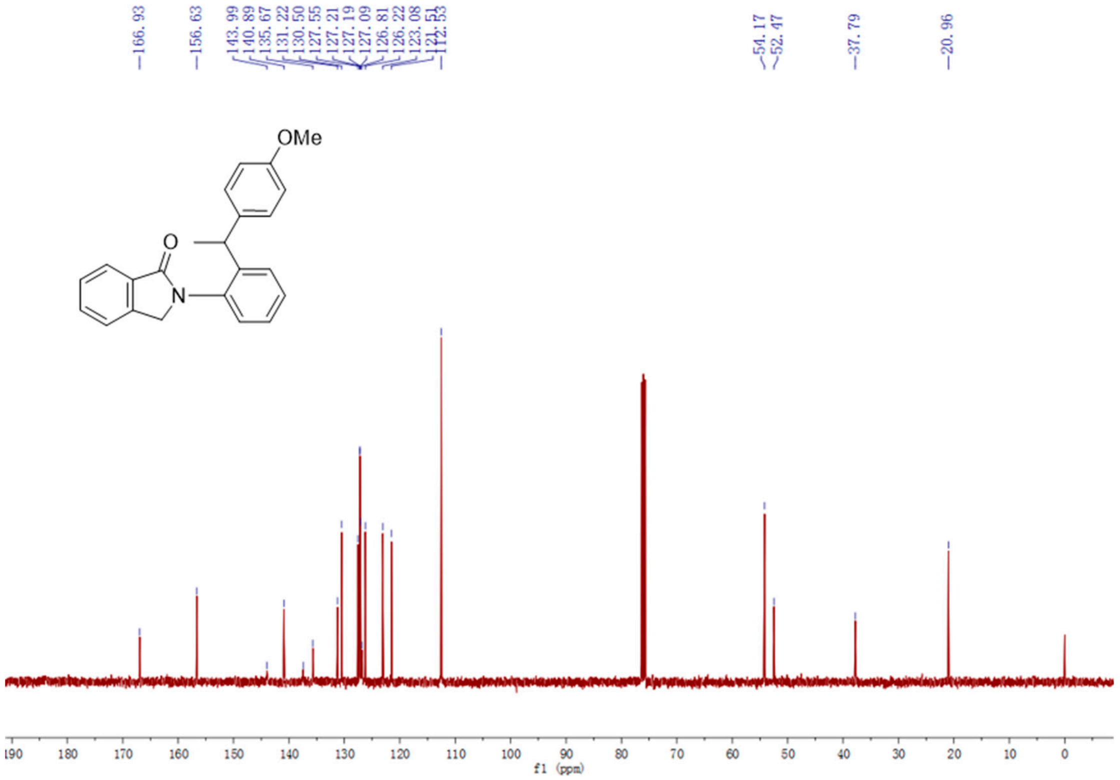

^{1}H NMR Spectrum of Compound **3f**

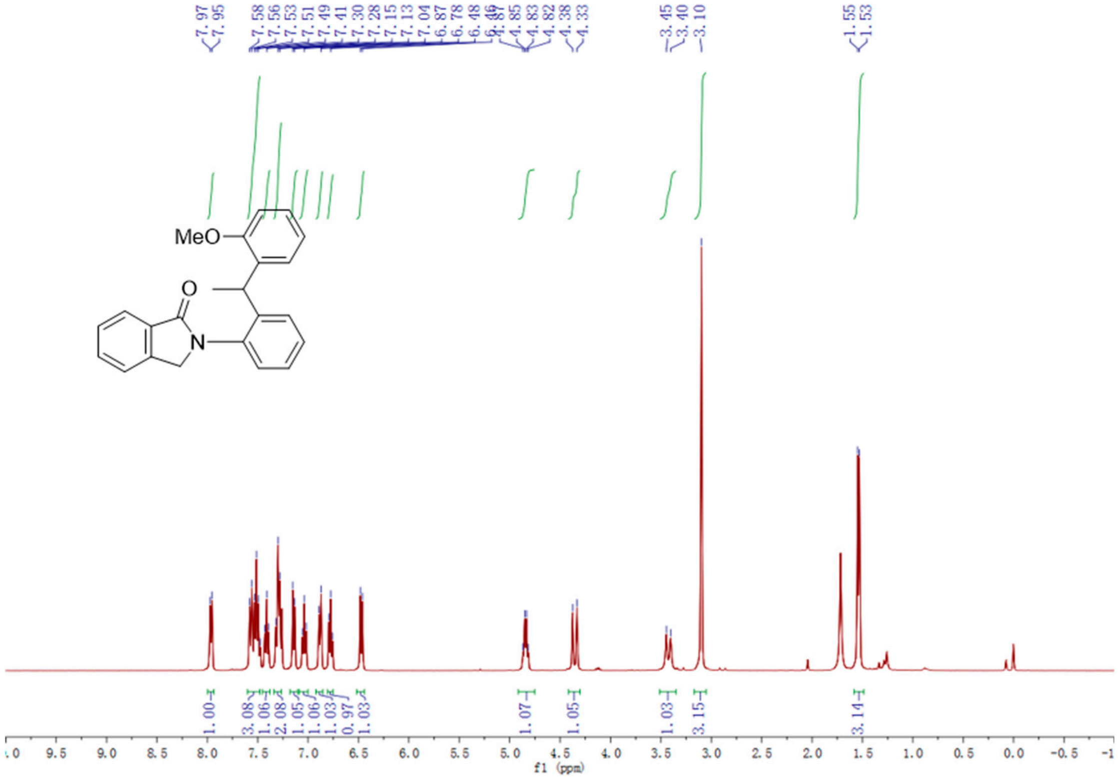

^{13}C NMR Spectrum of Compound **3f**

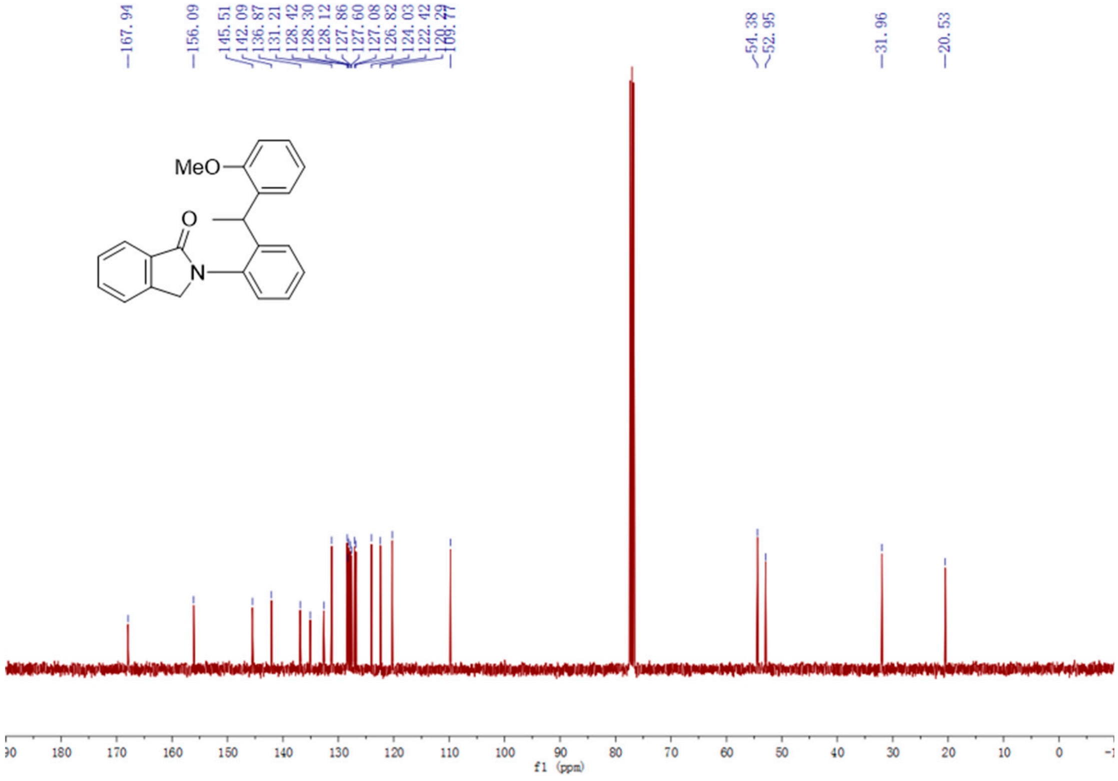

^{1}H NMR Spectrum of Compound **3g**

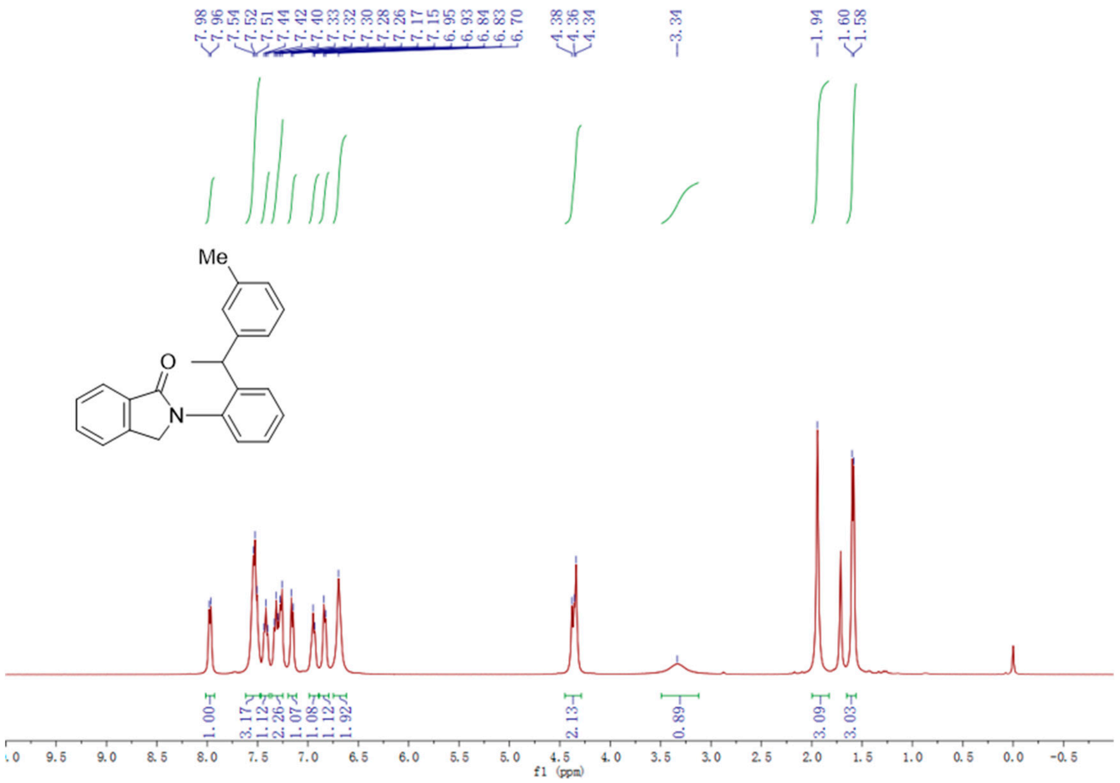

^{13}C NMR Spectrum of Compound **3g**

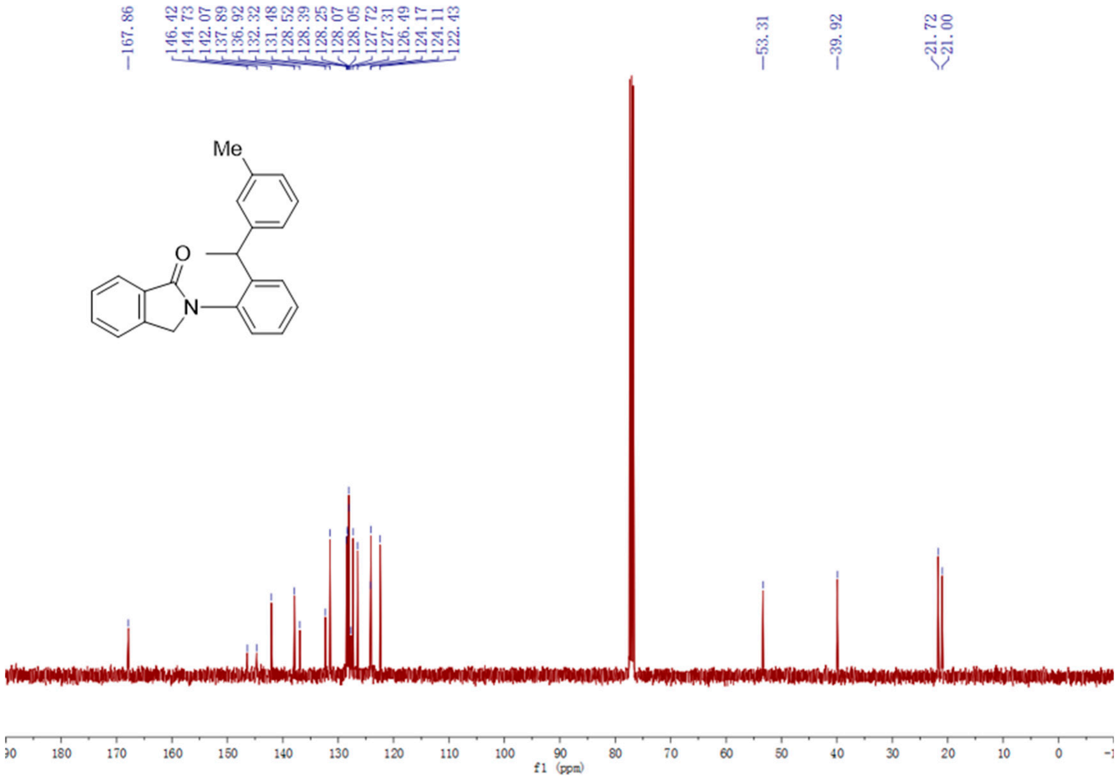

^{1}H NMR Spectrum of Compound **3h**

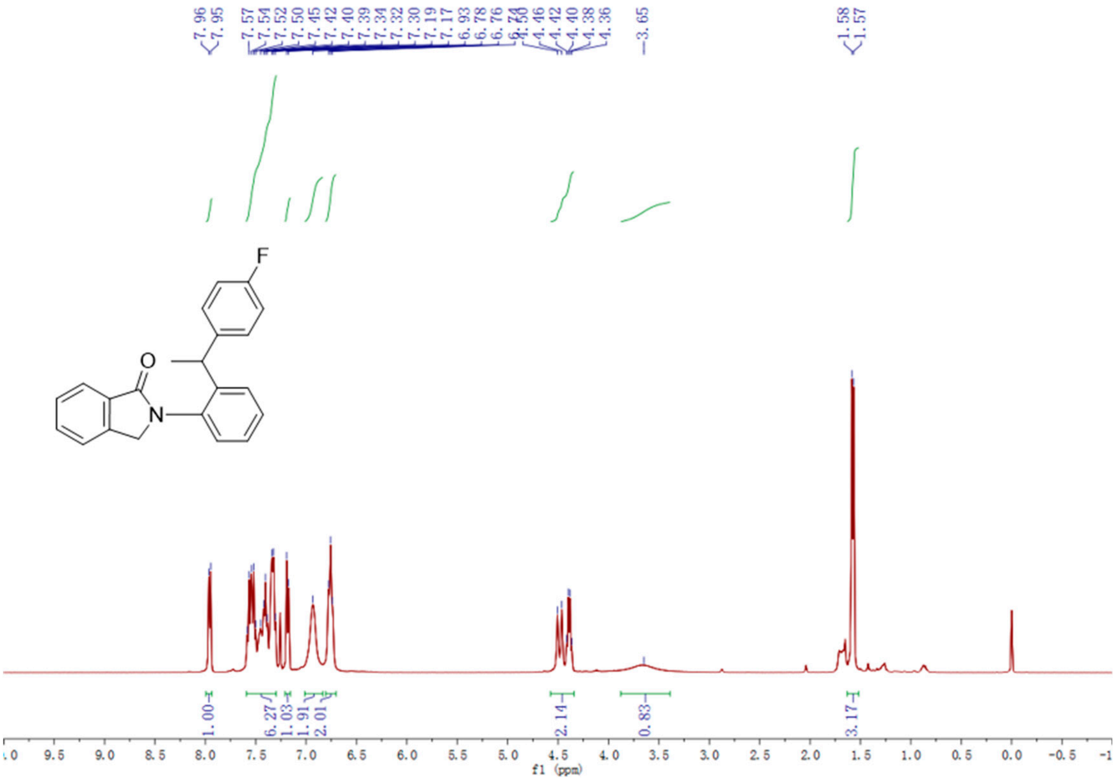

^{13}C NMR Spectrum of Compound **3h**

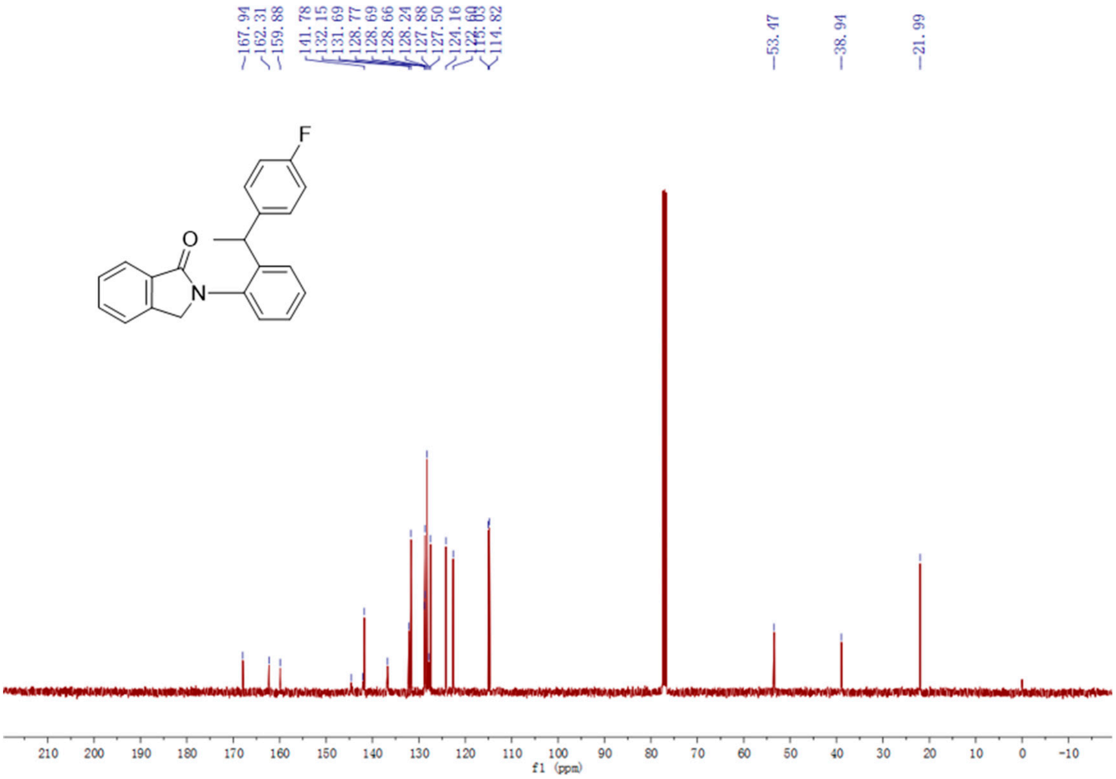

^{1}H NMR Spectrum of Compound **3i**

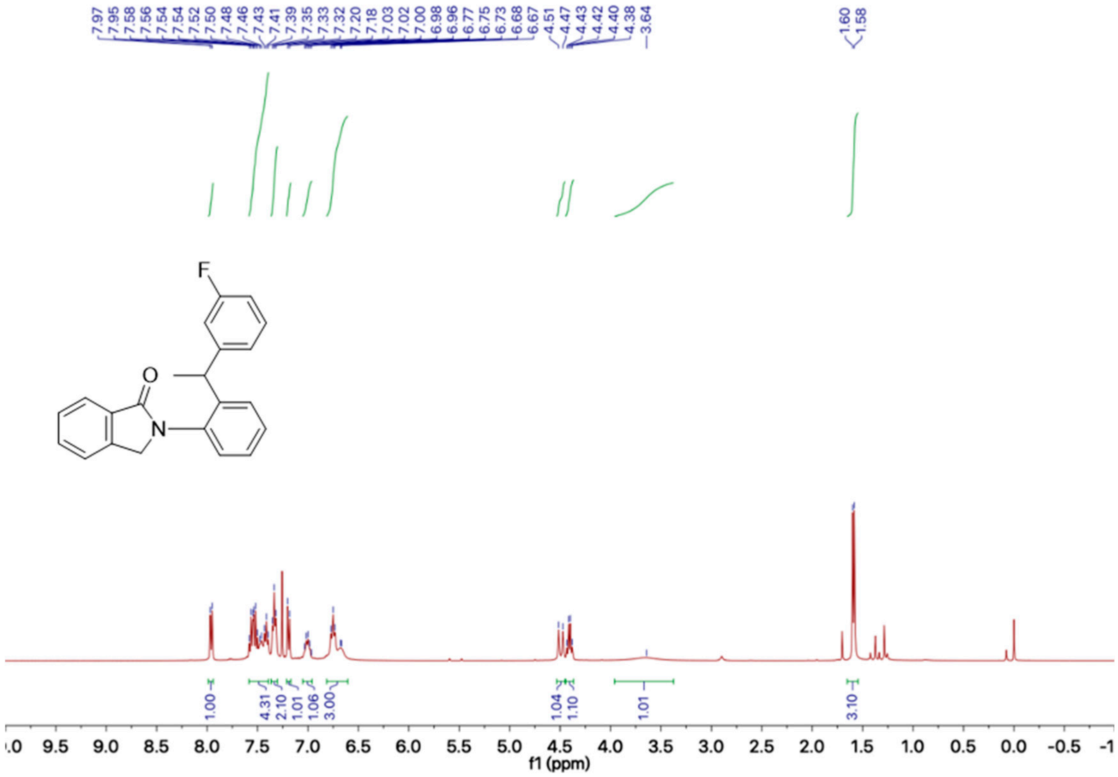

^{13}C NMR Spectrum of Compound **3i**

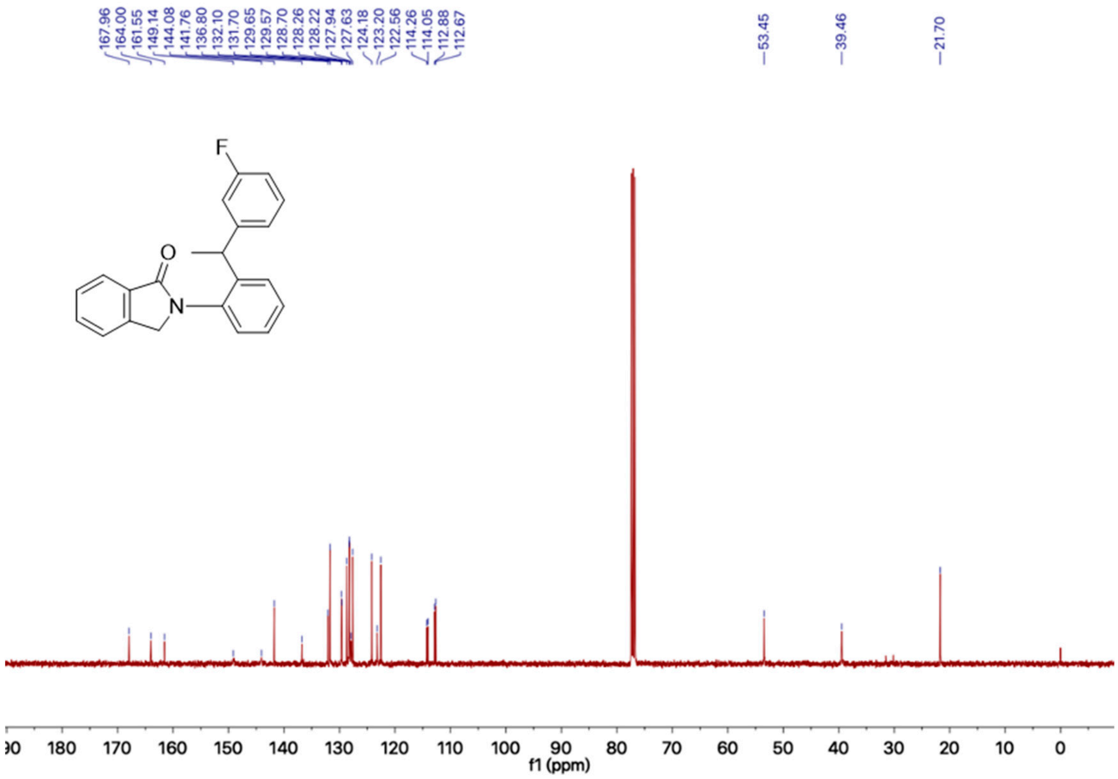

^{1}H NMR Spectrum of Compound **3j**

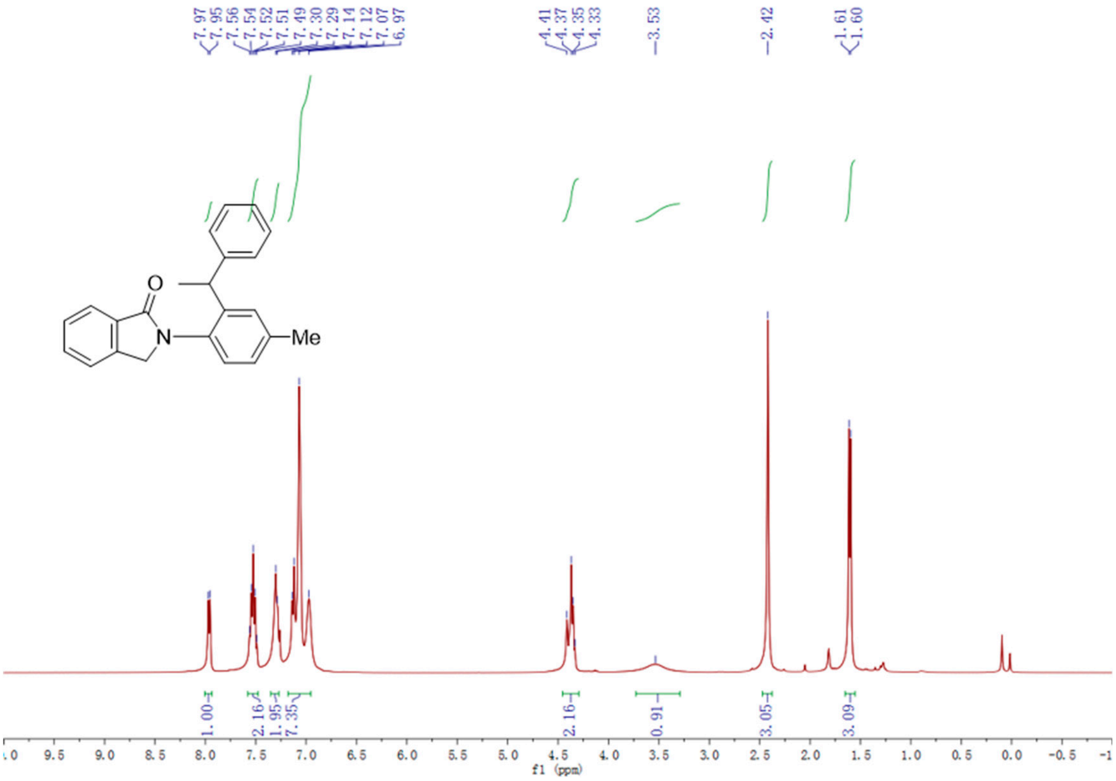

^{13}C NMR Spectrum of Compound **3j**

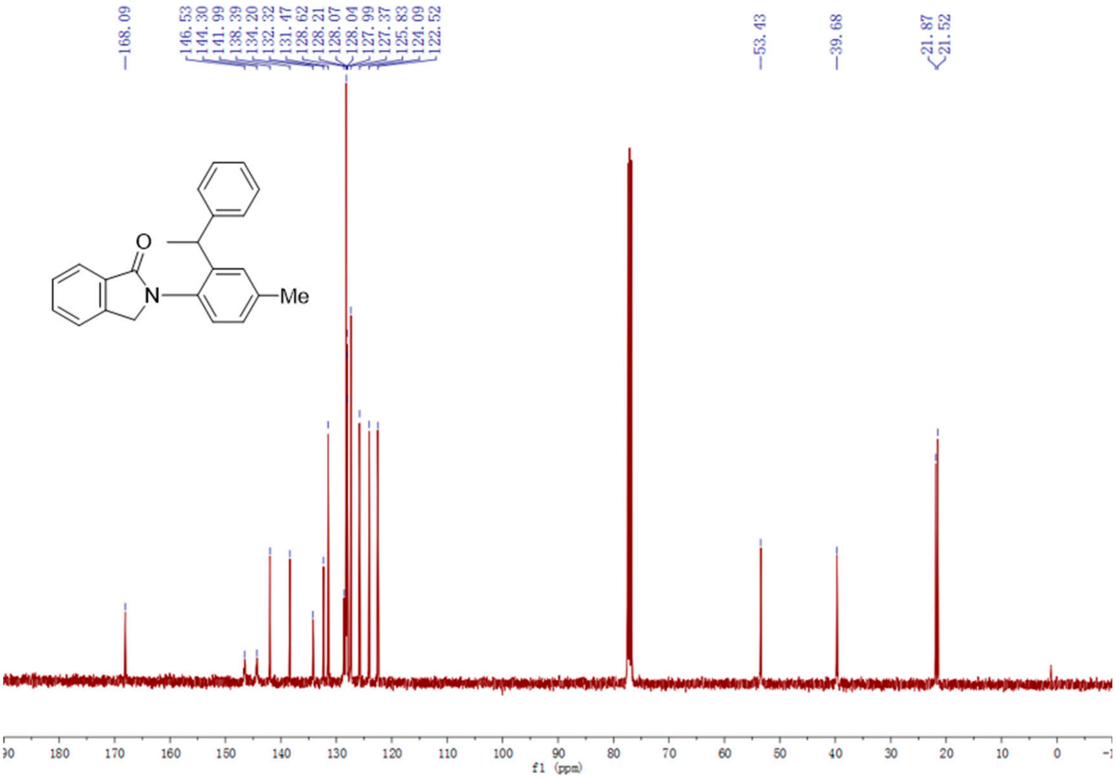

^{1}H NMR Spectrum of Compound **3k**

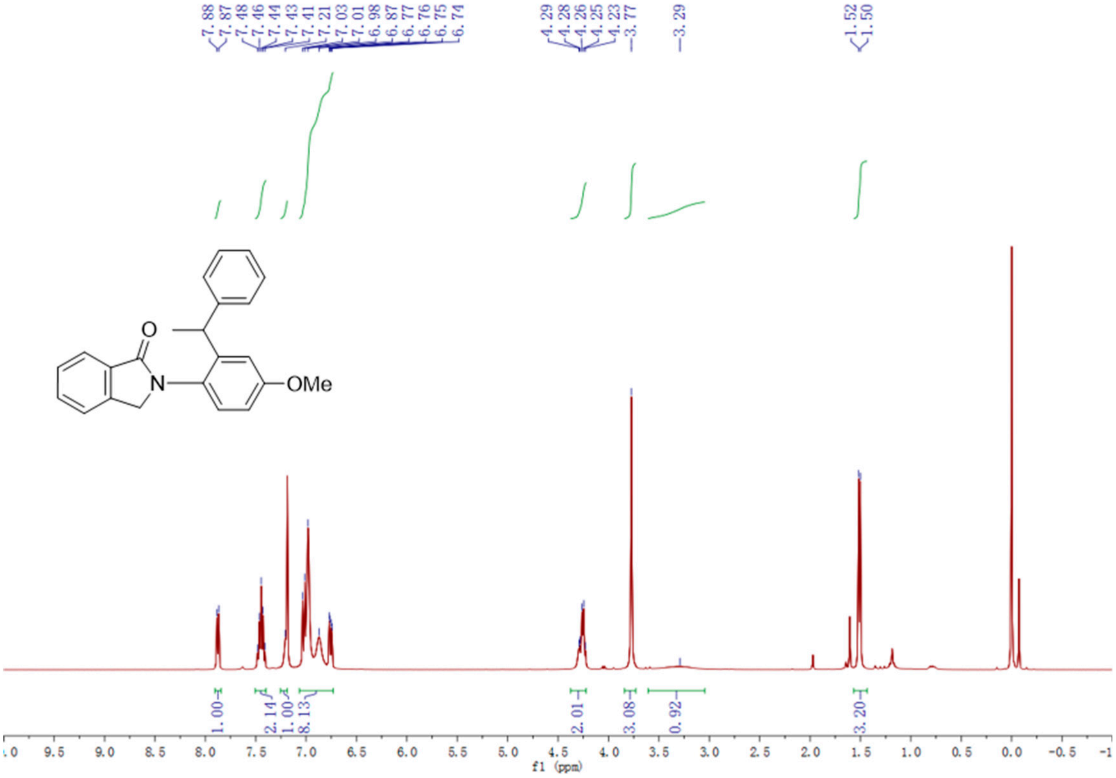

^{13}C NMR Spectrum of Compound **3k**

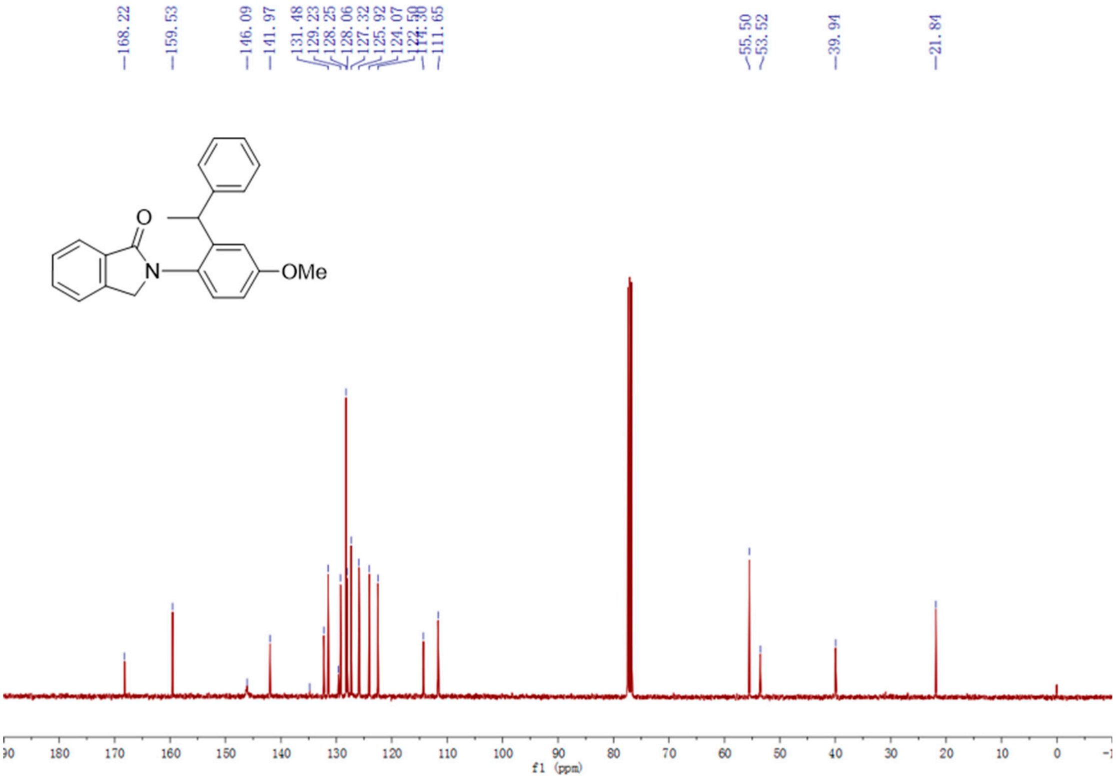

^{1}H NMR Spectrum of Compound **3l**

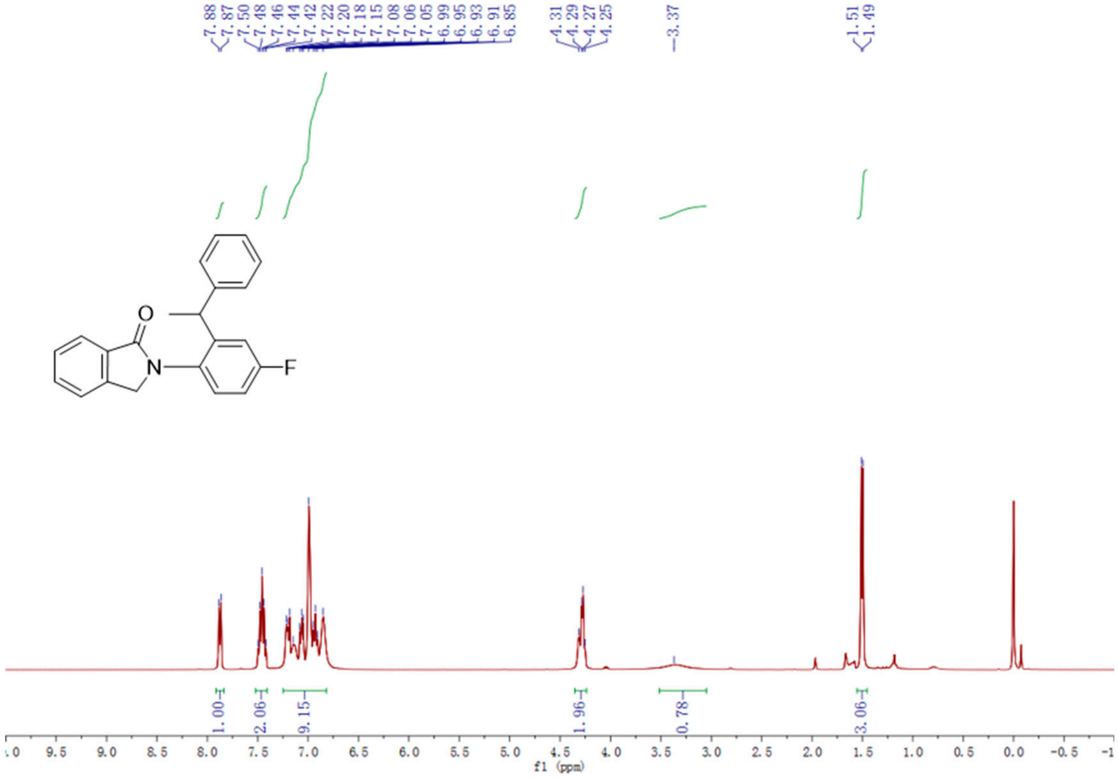

^{13}C NMR Spectrum of Compound **3l**

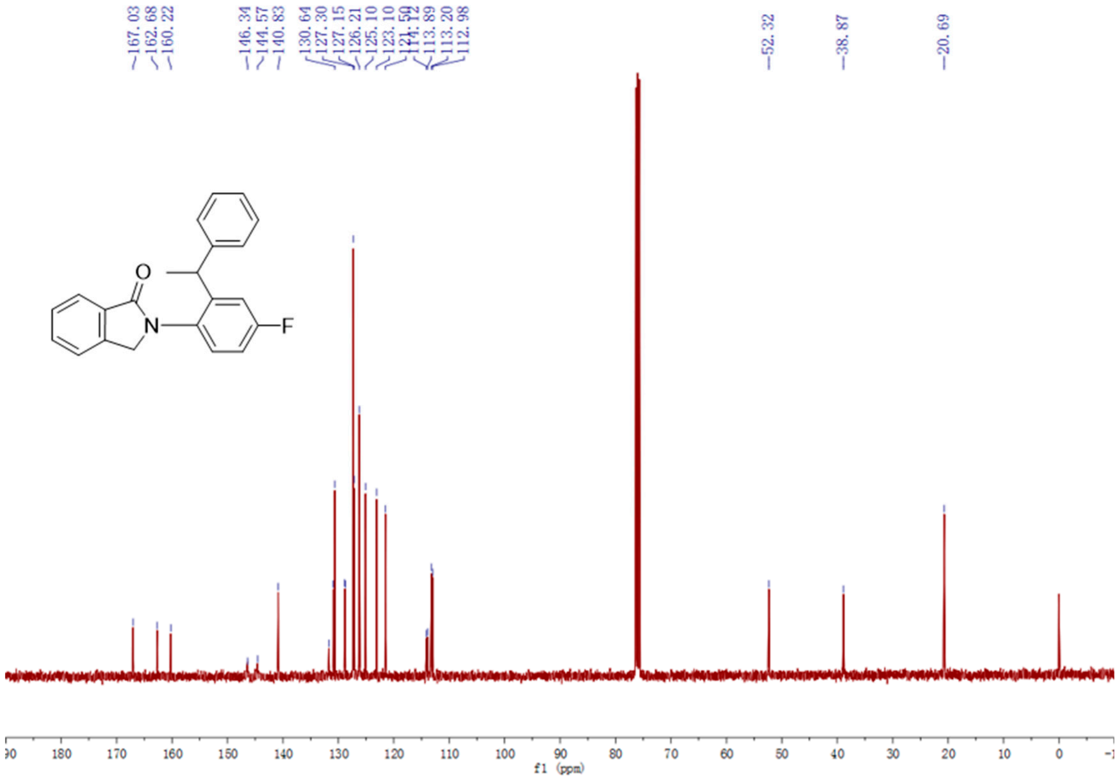

^{1}H NMR Spectrum of Compound **3m**

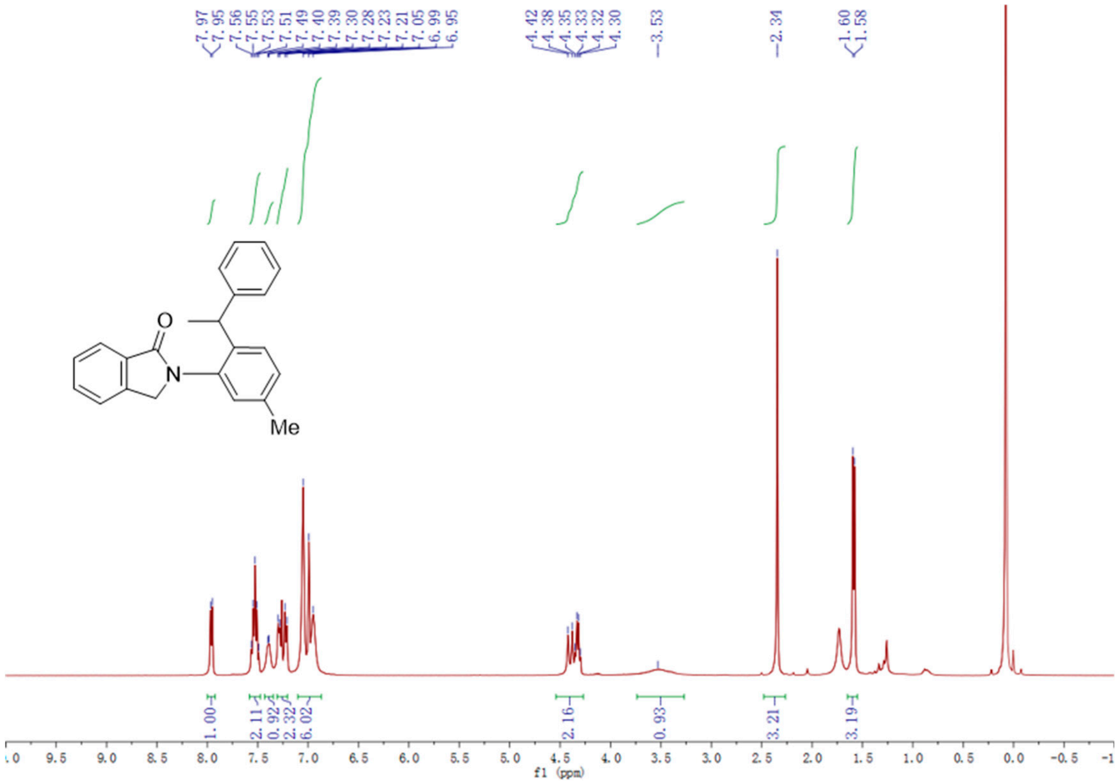

^{13}C NMR Spectrum of Compound **3m**

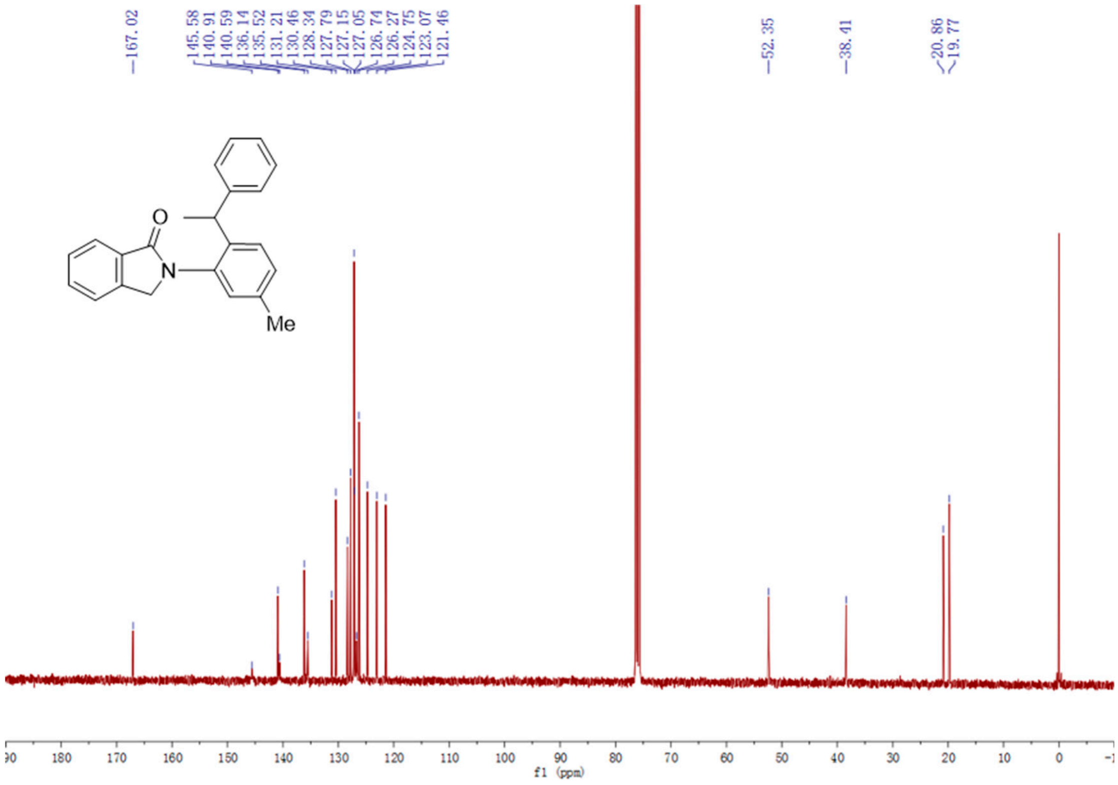

^{1}H NMR Spectrum of Compound **3n**

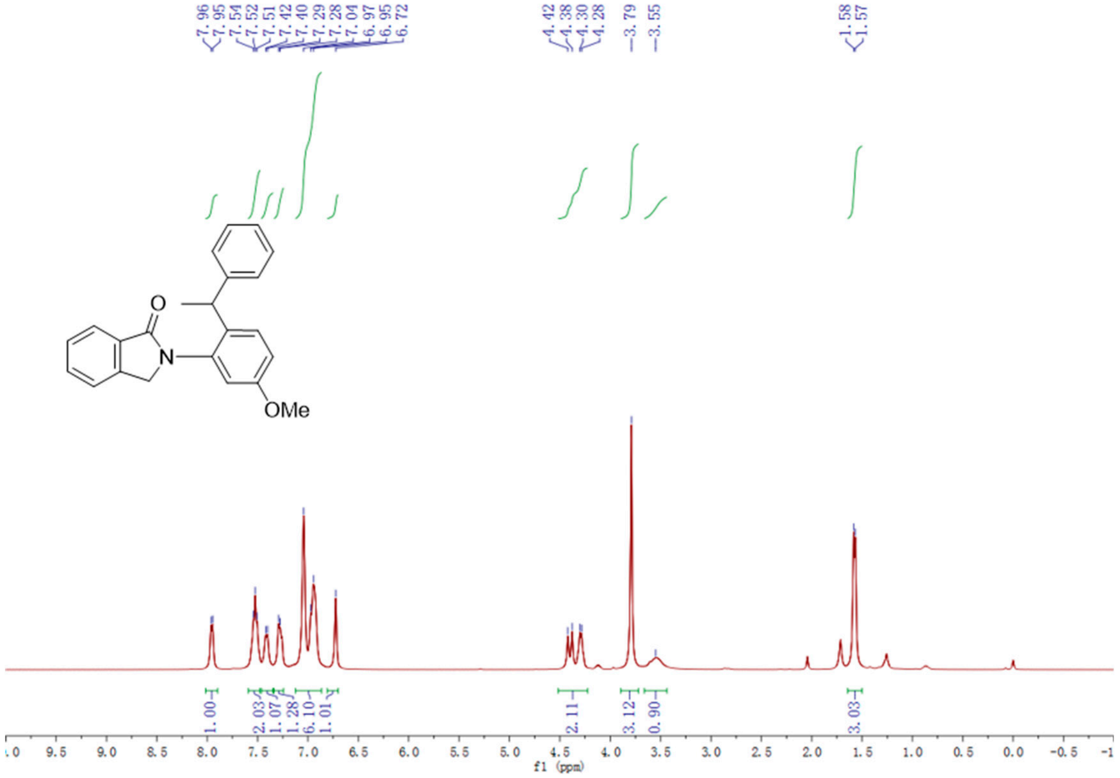

^{13}C NMR Spectrum of Compound **3n**

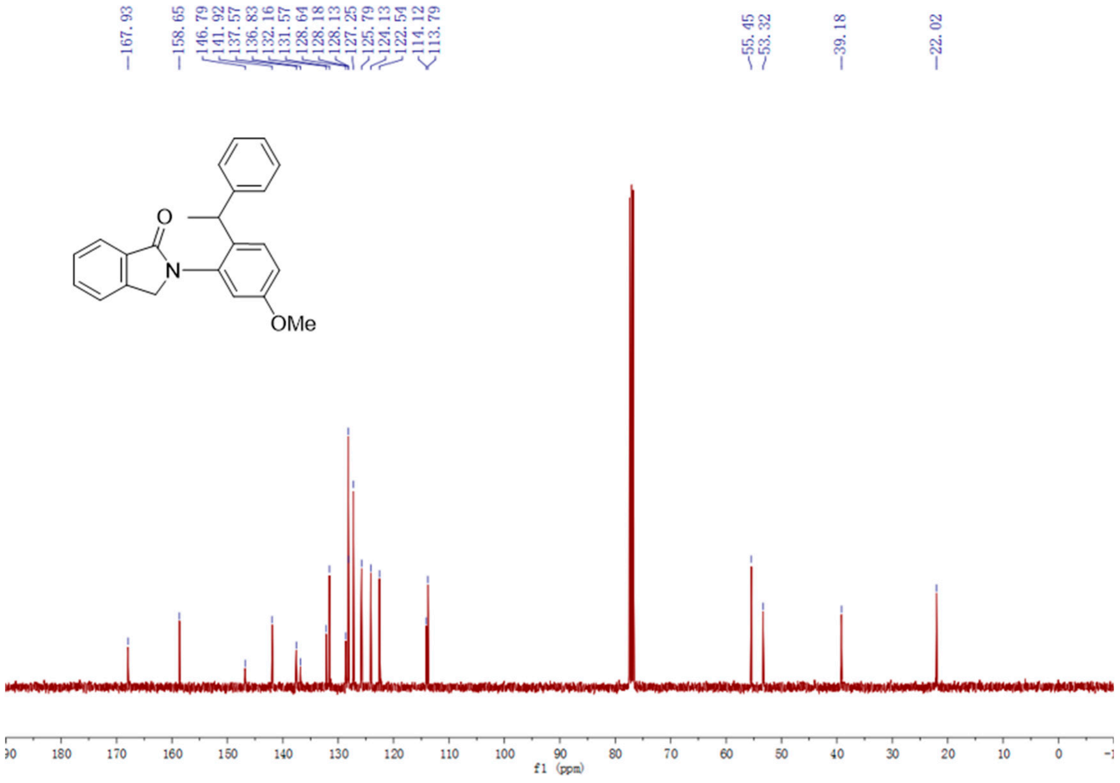

^{1}H NMR Spectrum of Compound **3o**

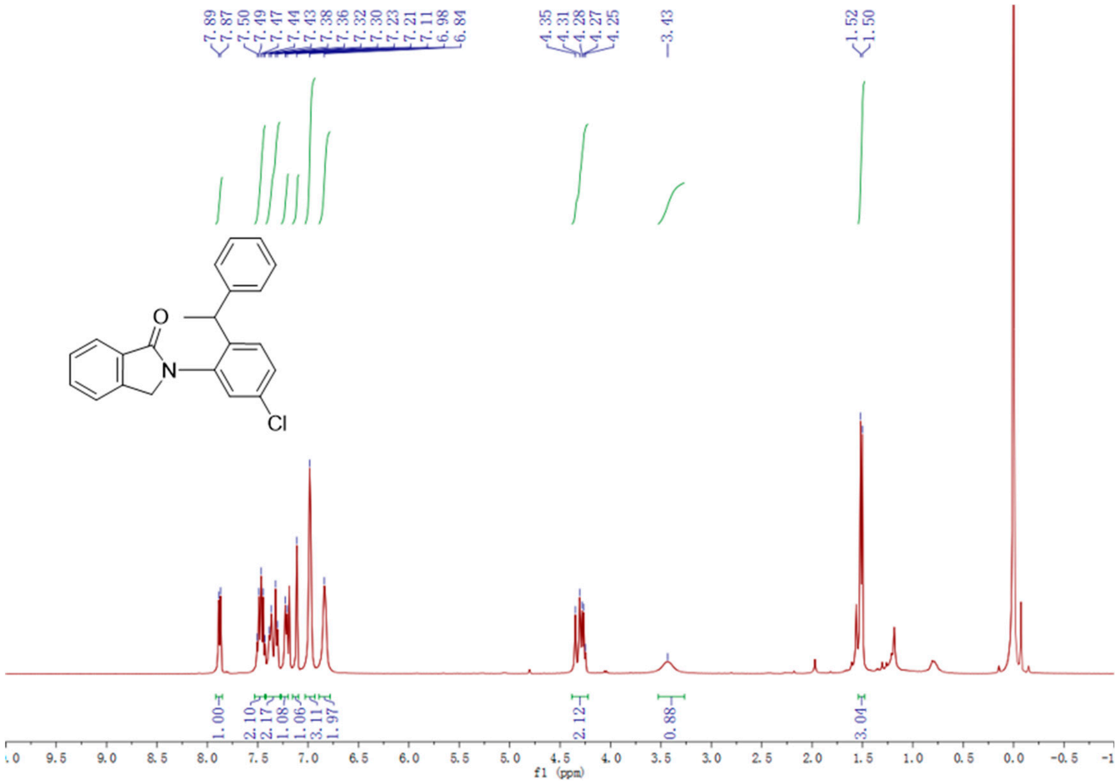

^{13}C NMR Spectrum of Compound **3o**

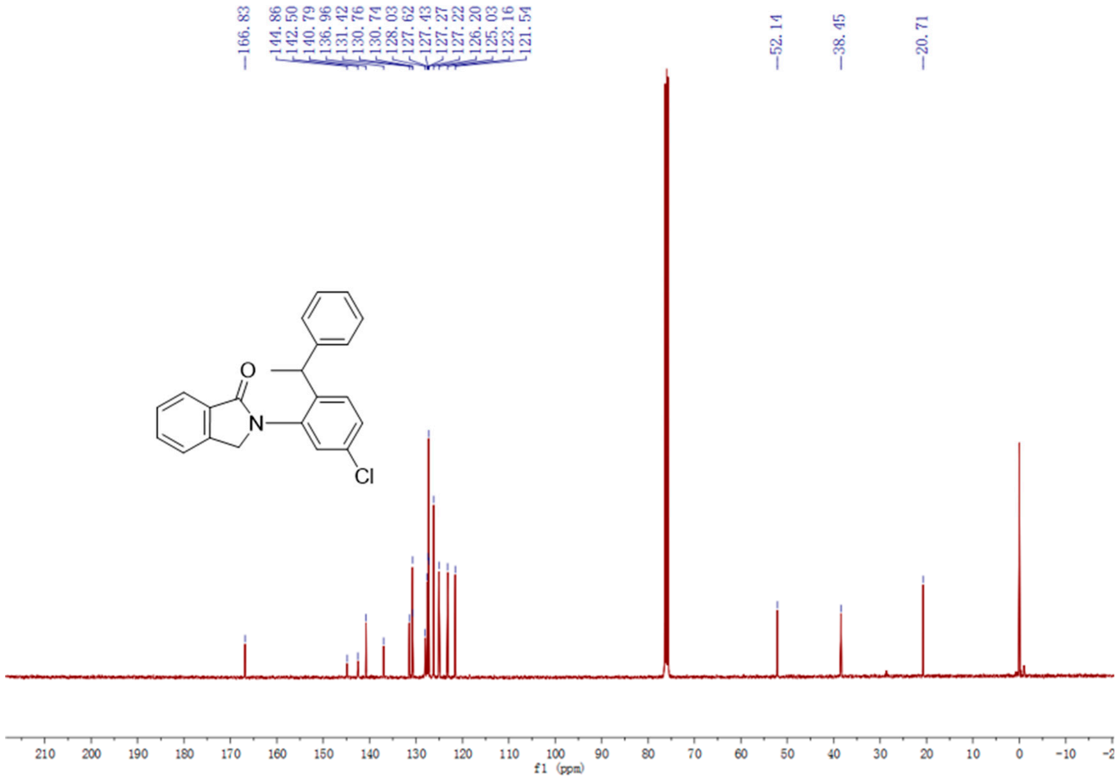

^{1}H NMR Spectrum of Compound **3p**

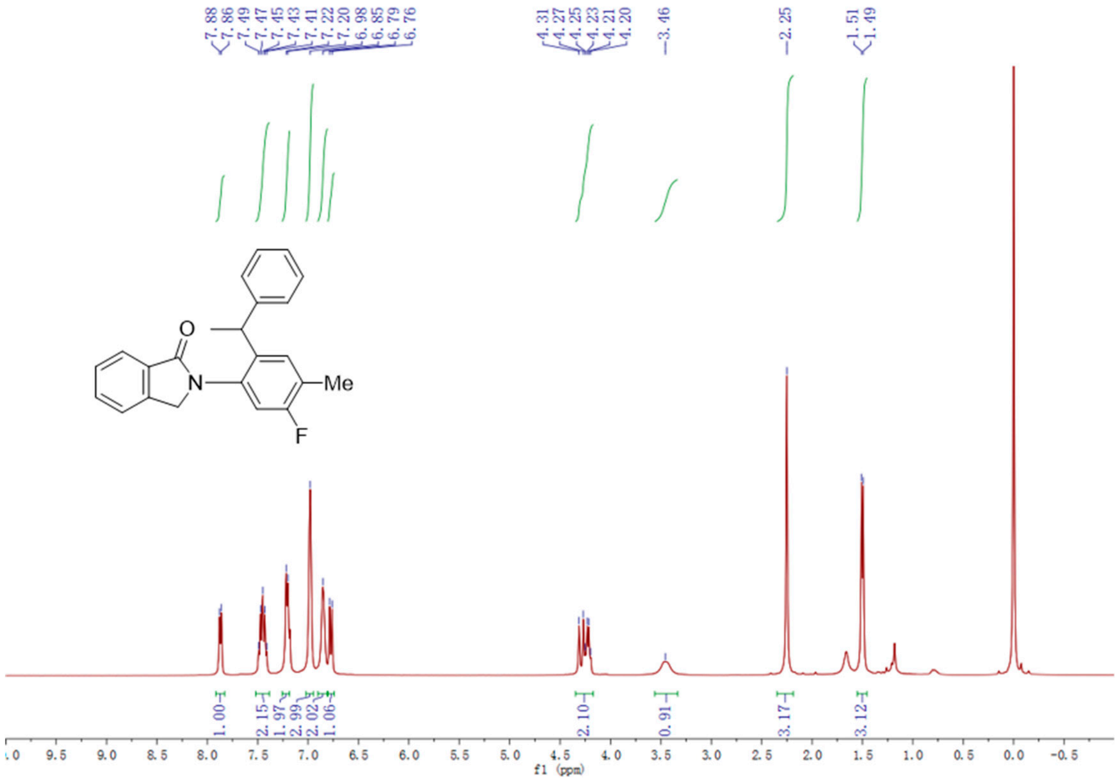

^{13}C NMR Spectrum of Compound **3p**

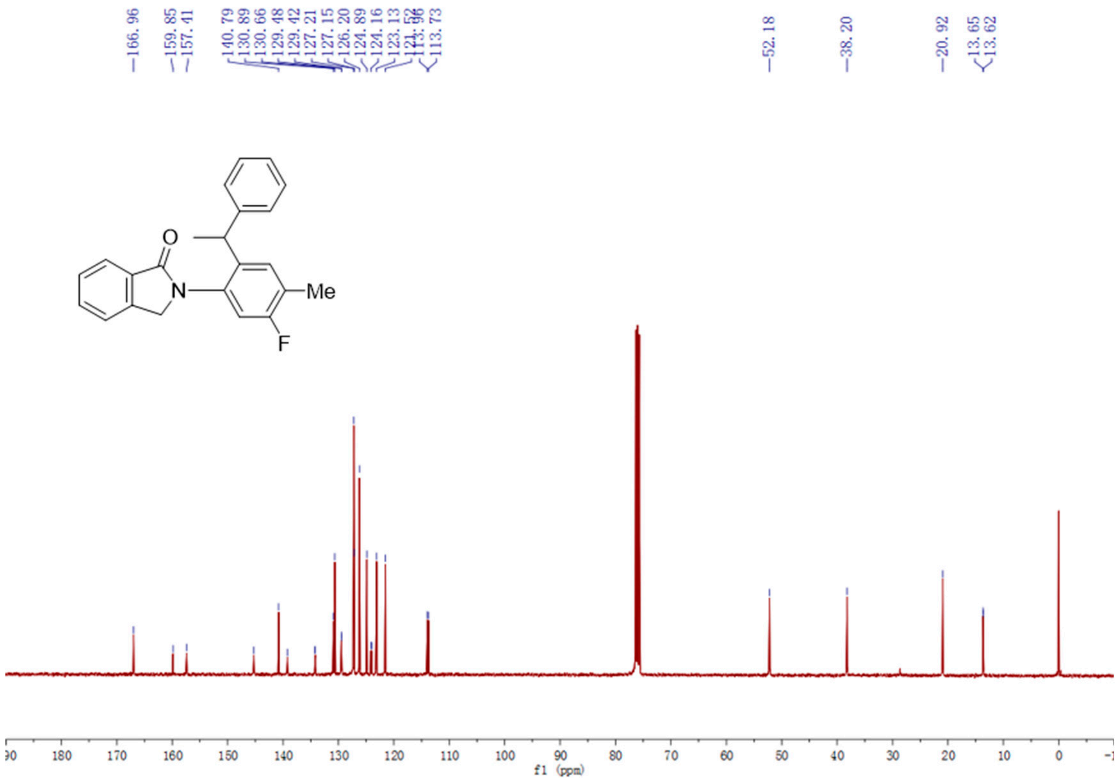

3. HPLC Spectra

HPLC spectra for compound *ent*-**3a** ((*R*)-L1)

Chiralcel IC column, *n*-hexane/*i*-PrOH = 100:6, flow rate = 1.0 mL/min, λ = 254 nm, t_R (major isomer) = 35.110 min, t_R (minor isomer) = 29.957 min.

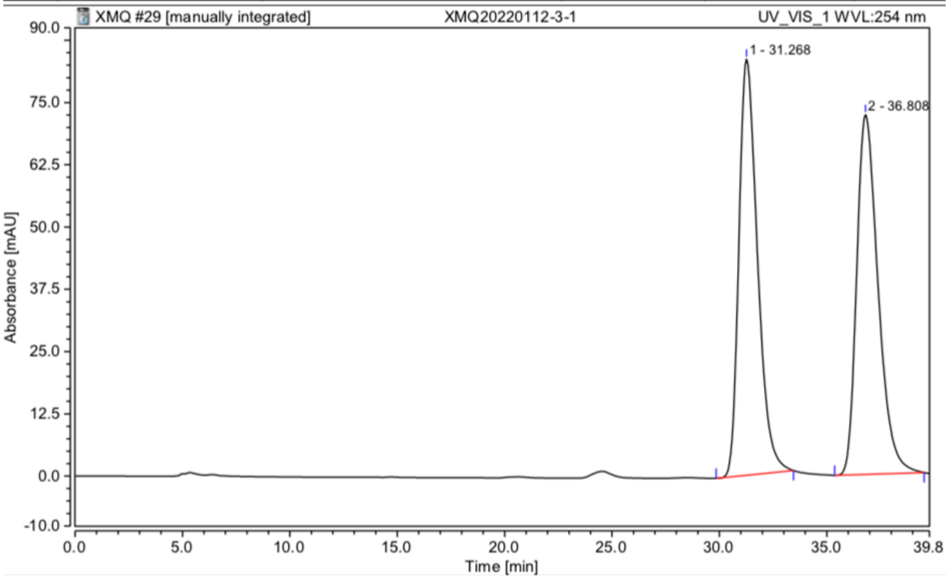

Integration Results						
No.	Peak Name	Retention Time min	Area mAU*min	Height mAU	Relative Area %	Amount n.a.
1		31.268	83.613	83.545	50.09	n.a.
2		36.808	83.321	72.234	49.91	n.a.
Total:			166.934	155.779	100.00	

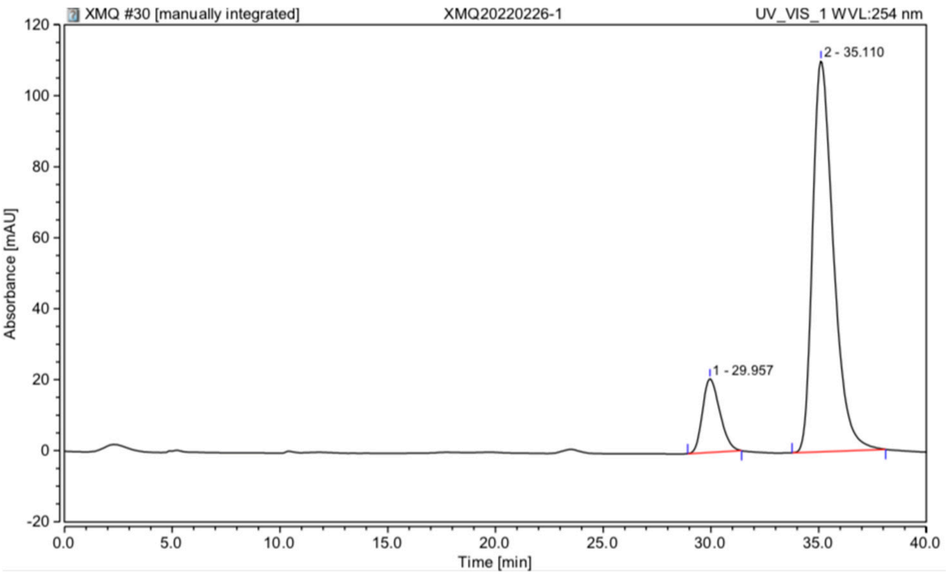

Integration Results						
No.	Peak Name	Retention Time min	Area mAU*min	Height mAU	Relative Area %	Amount n.a.
1		29.957	18.882	20.487	13.26	n.a.
2		35.110	123.481	110.165	86.74	n.a.
Total:			142.363	130.652	100.00	

HPLC spectra for compound *ent*-**3a** ((*S*)-L1)

Chiralcel IC column, *n*-hexane/*i*-PrOH = 100:10, flow rate = 1.0 mL/min, λ = 254 nm, t_R (major isomer) = 19.713 min, t_R (minor isomer) = 23.358 min.

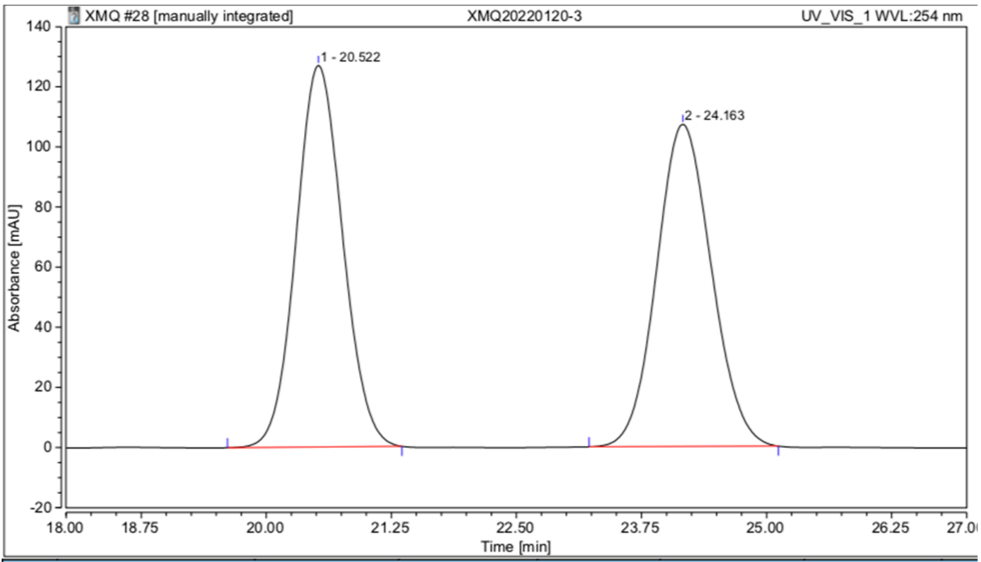

Integration Results

No.	Peak Name	Retention Time min	Area mAU*min	Height mAU	Relative Area %	Amount n.a.
1		20.522	68.535	126.902	50.16	n.a.
2		24.163	68.106	107.134	49.84	n.a.
**Total:**			**136.641**	**234.036**	**100.00**	

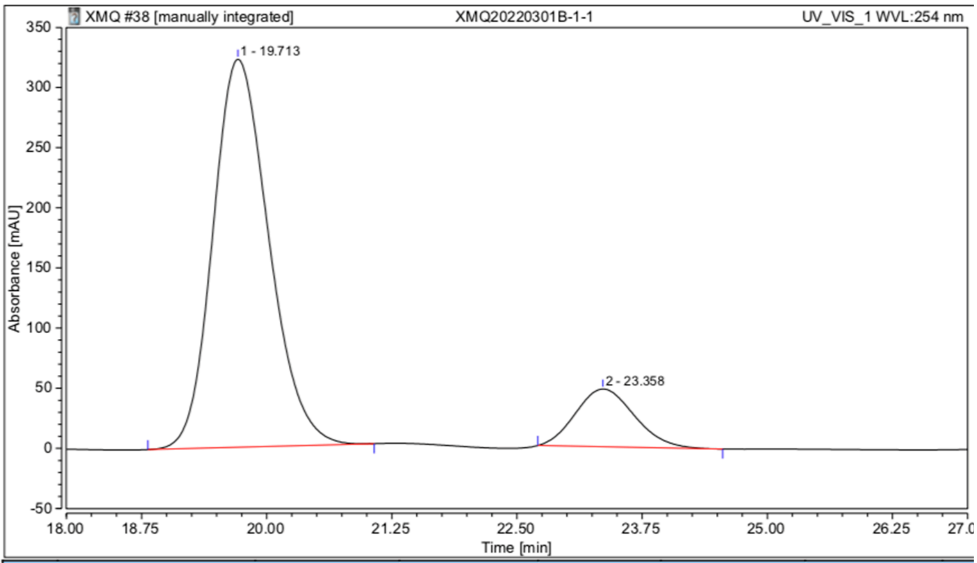

Integration Results

No.	Peak Name	Retention Time min	Area mAU*min	Height mAU	Relative Area %	Amount n.a.
1		19.713	208.528	322.494	86.45	n.a.
2		23.358	32.687	47.981	13.55	n.a.
**Total:**			**241.214**	**370.475**	**100.00**	

HPLC spectra for compound *ent*-**3c** ((*R*)-L1)

Chiralcel IC column, *n*-hexane/*i*-PrOH = 100:10, flow rate = 1.0 mL/min, λ = 254 nm, t_R (major isomer) = 19.801 min, t_R (minor isomer) = 20.807 min.

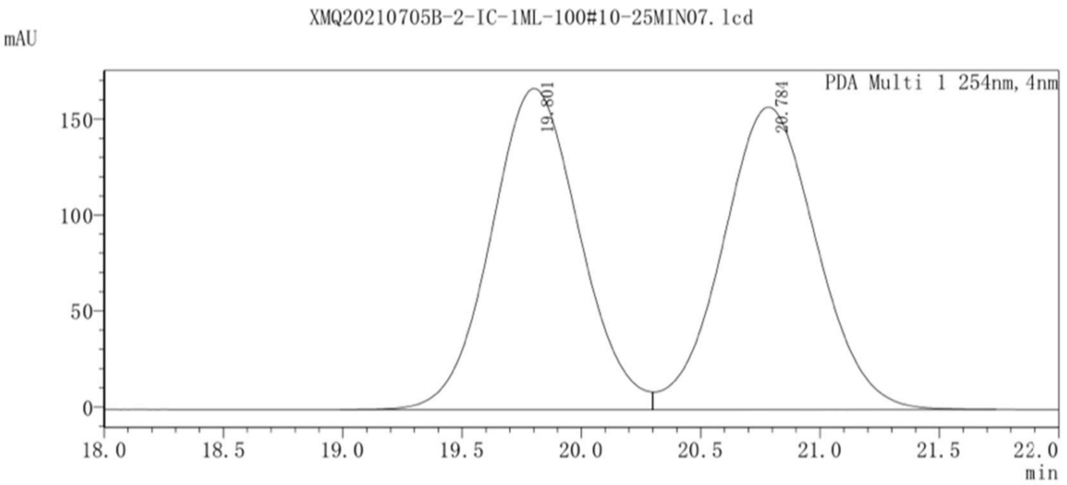

Peak No	Retention time	Area	Area%	Height
1	19.801	4310259	49.987	167211
2	20.784	4312423	50.013	157449
Total		8622682	100.00	324660

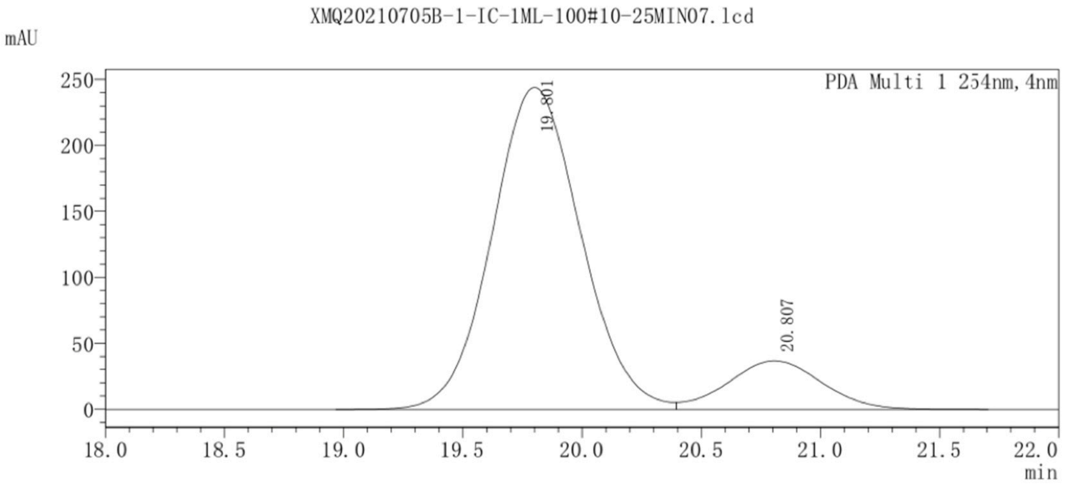

Peak No	Retention time	Area	Area%	Height
1	19.801	6329235	86.258	244163
2	20.807	1008338	13.742	36855
Total		7337572	100.00	281018
